# MXene Bioinks for 3D Bioprinting: Design and Translation

**DOI:** 10.1002/smll.74819

**Published:** 2026-07-28

**Authors:** Begüm Sarac, Seydanur Yücer, Fatih Ciftci

**Affiliations:** ^1^ Faculty of Engineering Department of Biomedical Engineering Fatih Sultan Mehmet Vakıf University Istanbul Turkey; ^2^ Biomedical Electronic Design Application and Research Center (BETAM) Fatih Sultan Mehmet Vakıf University Istanbul Turkey; ^3^ BioriginAI Research Group Department of Biomedical Engineering Fatih Sultan Mehmet Vakıf University Istanbul Turkey

**Keywords:** electrical percolation, electroactive hydrogels, MXene bioinks, rheological engineering, tissue regeneration, translational biofabrication

## Abstract

MXenes have emerged as electrically conductive, hydrophilic two‐dimensional nanomaterials uniquely suited for electroactive bioink development. However, the field lacks an integrated design framework that links MXene surface chemistry and colloidal behavior to printability, electrical percolation, and tissue‐specific biological outcomes. This review establishes a structure rheology biofunction paradigm for MXene‐based bioinks, critically examining how synthesis routes, surface terminations, and oxidation dynamics influence shear‐thinning behavior, network formation, and cytocompatibility. We analyze mechanistic pathways underlying cell–material interactions, including electrically mediated signaling and mechanotransduction in three‐dimensional constructs. Emerging in vivo studies are evaluated with emphasis on biodistribution, immune modulation, and long‐term biodegradation. Finally, we define key translational bottlenecks, oxidative instability, reproducibility, sterilization sensitivity, and regulatory classification, and propose actionable design criteria to advance MXene bioinks from laboratory constructs to clinically viable electroactive scaffolds.

## Graphical abstract

This study establishes a comprehensive framework for MXene‐based bioinks in 3D bioprinting, highlighting the interplay between rheological engineering and electroactive hydrogels. By optimizing electrical percolation and mediated signaling, these formulations drive tissue‐specific biological outcomes and accelerated regeneration. Ultimately, this approach bridges material design and translational biofabrication for advanced functional tissue engineering.

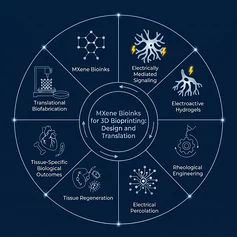

## 1 Introduction

Three‐dimensional (3D) bioprinting has emerged as a transformative platform in regenerative medicine, enabling the spatially controlled deposition of living cells and biomaterials to recreate complex tissue architectures. Beyond geometric precision, the next frontier of bioprinting lies in engineering bioactive microenvironments that recapitulate the biochemical, mechanical, and electrical cues governing native tissue function. While conventional hydrogel‐based bioinks provide cytocompatible and structurally supportive matrices, they remain inherently limited in electrical functionality and mechanical robustness. These constraints are particularly restrictive for electrically active tissues, including neural, cardiac, and skeletal muscle systems, where endogenous electrical signaling regulates mechanotransduction, lineage commitment, and functional maturation [1, 2].

Efforts to address these limitations have led to the incorporation of conductive nanomaterials into hydrogel bioinks. Carbon‐based fillers such as graphene and carbon nanotubes, as well as metallic nanoparticles, have demonstrated the ability to enhance conductivity and mechanical stiffness [3]. However, their clinical translation has been hindered by challenges including hydrophobic aggregation, poor aqueous dispersibility, inconsistent surface chemistry, and variable cytocompatibility. More importantly, the integration of conductivity often occurs at the expense of rheological performance or print fidelity, underscoring the need for materials that simultaneously satisfy electrical, mechanical, and processing requirements [4]. Efforts to address these limitations have led to the incorporation of conductive nanomaterials into hydrogel bioinks. Carbon‐based fillers such as graphene and carbon nanotubes, as well as metallic nanoparticles, have demonstrated the ability to enhance conductivity and mechanical stiffness. However, their clinical translation has been hindered by challenges including hydrophobic aggregation, poor aqueous dispersibility, inconsistent surface chemistry, and variable cytocompatibility. From a processing perspective, conductivity enhancement usually requires the formation of an interconnected conductive network above a critical filler concentration. Increasing filler loading can substantially alter hydrogel rheology by increasing viscosity, yield stress, and particle–polymer interactions, which may impair smooth extrusion, increase shear stress on encapsulated cells, or cause nozzle clogging. In addition, poorly dispersed conductive fillers can create agglomerates that disrupt homogeneous cross‐linking and produce irregular filament formation, reduced layer stacking, and loss of shape fidelity. Therefore, the integration of conductivity into bioinks is not simply an additive function but a formulation‐dependent trade‐off between electrical percolation, cytocompatibility, viscoelastic recovery, and print fidelity [5, 6, 7, 8].

In this context, MXenes, a rapidly expanding family of two‐dimensional transition metal carbides, nitrides, and carbonitrides, have emerged as uniquely positioned candidates for electroactive bioink engineering. Unlike many conventional conductive nanomaterials, MXenes combine metallic‐level conductivity with intrinsic hydrophilicity and tunable surface terminations (–O, –OH, –F), enabling stable colloidal dispersion within aqueous hydrogel systems [9]. This rare convergence of electrical percolation potential, surface reactivity, and aqueous processability positions MXenes not merely as conductive additives, but as design‐enabling nanocomponents capable of modulating bioink structure–property relationships [10].

Recent experimental studies have begun to demonstrate this potential across different tissue‐engineering contexts. Ti_3_C_2_‐based MXene nanosheets have been incorporated into hyaluronic acid/alginate, GelMA, GelMA/HAMA, and silicate‐modified hydrogel systems to produce electroconductive bioinks with improved printability, structural retention, cytocompatibility, and tissue‐specific bioactivity. These systems have been explored for osteogenic, myogenic, and neural applications, suggesting that MXene incorporation can influence not only electrical conductivity but also cellular adhesion, proliferation, differentiation, and functional maturation in printed constructs [8, 11, 12]. Nevertheless, these studies also reveal that MXene bioink performance is highly formulation‐dependent, with outcomes governed by nanosheet concentration, dispersion stability, hydrogel chemistry, cross‐linking strategy, and the balance between electrical percolation and biological compatibility [13].

Despite rapid growth in MXene‐based biofabrication studies, the field remains largely dominated by proof‐of‐concept demonstrations. Reported improvements in conductivity, stiffness, and cell viability are promising but often lack standardized benchmarking, systematic rheological characterization, or long‐term biological validation. Critical parameters, including oxidation dynamics, surface termination heterogeneity, nanosheet size distribution, and percolation thresholds, are inconsistently reported, limiting cross‐study comparability and obscuring structure–function relationships. Furthermore, in vivo evidence remains relatively sparse, and translational considerations such as sterilization stability, biodegradation kinetics, batch reproducibility, and regulatory classification are insufficiently addressed [14].

Addressing these challenges requires an integrative framework that connects MXene physicochemical characteristics with bioink rheology, print fidelity, cellular mechanobiology, and translational feasibility. Rather than treating electrical conductivity as an isolated performance metric, a holistic design paradigm is needed in which surface chemistry, colloidal behavior, network formation, and cellular signaling are evaluated as interdependent variables.

This review establishes a structure–rheology–biofunction–translation framework for MXene‐based bioinks in 3D bioprinting. Within this framework, MXene structural characteristics, including synthesis‐dependent physicochemical properties and surface chemistry, govern rheological behavior and printability. These rheological attributes subsequently influence biological performance, including cell–material interactions and tissue regeneration, while the combined physicochemical and biological properties ultimately determine translational feasibility and clinical applicability. We critically analyze how synthesis routes and surface terminations influence dispersion stability, shear‐thinning behavior, and electrically percolated network formation. We examine mechanistic insights into cell–material interactions, including integrin‐mediated adhesion, mechanotransduction, and electrically mediated signaling pathways in three‐dimensional constructs. Emerging in vivo studies are evaluated with emphasis on immune modulation, biodistribution, biodegradation, and functional integration. Finally, we identify key translational bottlenecks and propose actionable design criteria to guide the rational development of clinically viable electroactive bioinks [15].

## 2 MXenes in the Biomedical Context

MXenes constitute a rapidly expanding family of two‐dimensional (2D) transition metal carbides, nitrides, and carbonitrides described by the general formula M_n+1_X_n_T_x_, where M represents an early transition metal, X denotes carbon and/or nitrogen, and Tx corresponds to surface terminations such as ─O, ─OH, and ─F [16]. Beyond their compositional diversity, MXenes are distinguished by a unique electronic structure characterized by metallic conductivity originating from transition metal d‐band states, which enables high charge carrier density and efficient electron transport. When combined with their hydrophilic and chemically tunable surfaces, these electronic properties position MXenes as multifunctional nanoarchitectures rather than merely conductive fillers in biomedical systems.

Unlike many 2D nanomaterials that require extensive post‐functionalization to achieve aqueous processability, MXenes intrinsically exhibit colloidal stability due to their negatively charged surface terminations. This combination of metallic conductivity, hydrophilicity, and reactive surface chemistry establishes a physicochemical foundation particularly relevant for electroactive bioink engineering and other biomedical interfaces [17].

From a biomedical perspective, the relevance of MXenes arises from the ability of these physicochemical features to regulate biological interfaces. Their high electrical conductivity can support bioelectronic communication, electrophysiological signal transduction, and electrically mediated stimulation, which are particularly important for neural, cardiac, and skeletal muscle tissues. At the same time, their hydrophilic surface terminations provide anchoring sites for polymer chains, biomolecules, peptides, growth factors, and therapeutic agents, enabling their integration into hydrogels, coatings, scaffolds, and implantable or wearable biointerfaces [17, 18, 19, 20]. Accordingly, MXene‐based biomaterials have been explored in biosensing, drug delivery, photothermal cancer therapy, antibacterial treatment, wound healing, and tissue engineering, demonstrating that MXenes can act as multifunctional biomedical components rather than passive conductive additives [21, 22, 23].

However, the biomedical performance of MXenes is highly context‐dependent. Their interactions with cells and tissues are governed by composition, lateral flake size, surface termination chemistry, oxidation state, concentration, exposure duration, and the surrounding matrix. While many MXene‐containing systems exhibit favorable cytocompatibility and bioactivity at optimized concentrations, excessive loading or poorly controlled degradation may induce membrane disruption, oxidative stress, inflammatory responses, or altered cell viability. Therefore, evaluating MXenes in biomedical systems requires not only electrical and mechanical characterization but also systematic assessment of hemocompatibility, immune response, biodegradation behavior, biodistribution, and long‐term biosafety [24, 25, 26]. This issue is particularly important for MXene‐based bioinks, where nanosheets are placed in direct contact with living cells during printing and remain embedded within three‐dimensional tissue‐like constructs after fabrication.

### 2.1 Structure and Surface Chemistry

MXenes are typically synthesized through the selective etching of the A‐layer from layered MAX phases, resulting in ultrathin nanosheets with high specific surface area and abundant surface functionalities. This topochemical transformation not only generates atomically thin lamellae but also introduces defect sites, heterogeneous termination distributions, and edge‐reactive domains that collectively influence interfacial behavior. The resulting nanosheets possess high aspect ratios, which play a critical role in reducing electrical percolation thresholds when incorporated into hydrogel matrices, an important determinant of conductivity in bioinks.

Surface terminations (─O, ─OH, ─F) introduced during etching significantly modulate hydrophilicity, zeta potential, and surface energy. These terminations are not uniformly distributed; basal planes and edge sites often exhibit different chemical environments, with edge sites being more susceptible to oxidation and structural transformation. The relative proportion of ─O, ─OH, and ─F groups directly influences colloidal thermodynamics, dispersion stability, and electrostatic interactions with biomolecules. For instance, higher ─O/─OH termination densities generally enhance hydrogen bonding with aqueous media, improving dispersion stability, whereas fluorine‐rich surfaces may alter protein adsorption profiles and potentially necessitate secondary modification to optimize biocompatibility [17].

These surface characteristics ultimately dictate how MXenes interact with biological environments, which is closely linked to their oxidative stress–related cellular effects. The biological effects of Ti_3_C_2_ MXenes are largely related to oxidative stress mechanisms. In vivo and in vitro studies have shown that MXenes can trigger inflammatory responses and cell membrane damage by increasing the formation of reactive oxygen species (ROS). A strong correlation has been found between toxicity and the oxidative potential of MXenes, and the reduction of toxic effects upon application of N‐acetylcysteine, a ROS scavenging agent, confirms that oxidative stress is one of the key mechanisms. These findings suggest that MXene biocompatibility depends not only on the presence of the material but also on its interactions with biological processes such as oxidative stress and inflammation [27]. Notably, these oxidative stress effects are closely linked to MXene surface properties, which determine their cellular uptake and subsequent intracellular damage.

The biocompatibility and toxicity of MXene are closely related to their surface properties. Studies on Ti_3_C_2_ and Ti_3_CN MXene have shown that MXene with unaltered surfaces are more readily taken up by cells, resulting in excessive ROS production, mitochondrial damage, lysosomal degradation, and apoptosis. Conversely, surface alterations resulting from oxidative senescence reduced cellular uptake and oxidative stress, thereby decreasing cytotoxicity. These findings suggest that MXene biocompatibility is determined by surface reactivity, cell‐material interactions, and oxidative stress mechanisms [28]. In addition to cellular toxicity mechanisms, MXene nanostructures show size‐dependent effects that extend to immune response and biodegradation processes. MXene quantum dots (MQDs), depending on their size and surface engineering, can interact with the immune system, modulating T‐cell activity and immune response. They also exhibit controlled degradation kinetics and biodegradation behavior, displaying temporary biopersistence rather than chronic accumulation. These characteristics suggest that the systemic compatibility, immune response, and biodegradation processes of MQDs should be considered together [29]. Collectively, these findings indicate that MXene biocompatibility arises from coupled physicochemical surface properties and biological mechanisms including oxidative stress, immune modulation, and degradation behavior.

Different etching protocols, including fluorine‐based and fluorine‐free strategies, further affect termination chemistry and defect density. Fluorine‐containing etchants tend to increase –F terminations, which may modify surface charge distribution and influence subsequent biointerface interactions. Such synthetic variability introduces batch‐dependent heterogeneity, complicating cross‐study comparison and underscoring the importance of standardized physicochemical characterization.

Due to their hydrophilic surfaces and high negative surface charge, MXenes initially demonstrate favorable aqueous dispersibility, facilitating homogeneous integration into hydrogel networks and bioinks. However, under physiological conditions, particularly in oxygen‐rich aqueous environments, rapid oxidation can occur. Delaminated Ti_3_C_2_T_x_ nanosheets are especially susceptible to oxidation at edge sites, leading to gradual transformation into TiO_2_‐rich domains. This oxidative conversion can reduce electrical conductivity by several orders of magnitude and alter zeta potential, thereby impacting both percolation behavior and protein adsorption dynamics.

Oxidation‐driven structural evolution not only compromises electroactivity but also modifies biological identity by reshaping surface chemistry and redox behavior. Consequently, long‐term stability in physiological environments remains a central challenge for translational applications. To mitigate premature degradation and aggregation, various surface engineering strategies, including polymer grafting with PEG, hyaluronic acid (HA), or protein coatings, have been explored [30]. These modifications provide steric stabilization, reduce oxidative susceptibility, and modulate nano–bio interactions by altering hydration layers and surface accessibility.

In addition to passive stabilization strategies, MXene systems have been designed to actively modulate oxidative stress in biological environments. Ren et al. developed a ROS‐scavenging MXene‐based conductive hydrogel by integrating Ti_3_C_2_ MXene nanosheets into a temperature‐sensitive ECM matrix. The system reduces intracellular reactive oxygen species through the intrinsic redox activity of MXene, thereby mitigating oxidative stress and protecting cardiomyocytes from apoptosis. In addition, the conductive network enhances intercellular electrical signaling via increased Connexin43 (Cx43) expression, indicating that MXene‐mediated redox regulation and surface reactivity contribute to both electrochemical and biological stabilization in oxidative environments [31].

Alongside antioxidant‐based biological strategies, polymer encapsulation offers a complementary physicochemical approach to improving MXene stability. Kumar et al. demonstrates a polymer encapsulation strategy to enhance the oxidative stability of Ti_3_C_2_T_x_ MXene‐based sensors. By embedding MXene within a siloxane‐based polymer matrix, the system effectively reduces environmental degradation, leading to a more than twofold increase in device half‐life. The encapsulation not only improves structural stability but also preserves and even enhances sensing performance through interfacial chemical interactions. These results highlight polymer encapsulation as an effective approach to mitigate oxidation‐induced instability in MXene‐based devices. Compared with antioxidant‐based approaches, polymer encapsulation offers superior physical and environmental protection, making it more suitable for device stability; however, it lacks direct bioactive or redox‐regulating functions in physiological systems [32].

Beyond bulk‐level physical encapsulation strategies, interfacial engineering approaches have also been explored to further regulate MXene surface reactivity and enhance long‐term stability through controlled surface passivation Wang et al. investigates the controlled adsorption of quaternary ammonium ions on the Ti_3_C_2_ MXene surface under an external electric field (EEF), elucidating the surface passivation mechanism. DFT simulations showed that the EEF effectively modulated the ion adsorption energy and the degree of surface encapsulation. In particular, the formation of Ti─O─N bonds resulted in a denser protective layer, reducing MXene surface reactivity and enhancing surface passivation. This process improved electrochemical stability and ensured high capacitance conservation over a long cycle life. These results demonstrate that electric field‐assisted surface passivation is an effective strategy for improving MXene stability. In contrast to bulk encapsulation, surface passivation provides more precise interfacial control and improved electrochemical durability, although its effectiveness is often dependent on external stimuli such as electric fields [33].

Beyond electrochemical stabilization strategies focused on interfacial surface passivation, MXene‐based systems have also been extended to biomedical applications, where similar redox‐regulating principles are employed to reconstruct pathological microenvironments and promote tissue regeneration. Zhu et al. reported a MXene‐containing conductive hydrogel for spinal cord injury repair, where the MXene component plays a key role in regulating local redox chemistry by lowering ROS levels and modulating macrophage polarization toward an anti‐inflammatory phenotype. This redox balancing effect, combined with MXene's surface‐related conductivity, creates a more stable microenvironment that supports neural regeneration. Importantly, the system highlights how MXene surface chemistry and electron transfer properties can indirectly regulate oxidative stress in biological systems [34]. These MXene stabilization strategies, together with their mechanisms, advantages, and limitations, are systematically compared in Table 1. Overall, integrating physical stabilization through encapsulation, interfacial engineering via surface passivation, and biological redox modulation using antioxidant and microenvironment strategies appears to be the most effective approach for achieving long‐term MXene stability in biomedical applications.

**TABLE 1:** Comparison of MXene stabilization strategies for oxidative stability in biomedical applications.

Strategy	Mechanism	Main advantage	Limitation	Refs.
PEG HA protein coatings	Steric and hydration layer stabilization	Improved biocompatibility and dispersion	Limited long term oxidative protection	[30]
Antioxidant incorporation	Reactive oxygen species scavenging	Reduced oxidative stress and cellular protection	Finite activity over time	[31]
Polymer encapsulation	Physical barrier against oxygen and moisture	Strong environmental and structural stability	Reduced bioactivity	[32]
Surface passivation	Interfacial bonding and ion adsorption control	Enhanced electrochemical stability	Often stimulus dependent	[33]
Redox microenvironment engineering	Regulation of oxidative and inflammatory signals	Promotes tissue level regeneration	High system complexity	[34]

MXenes, derived from MAX phases composed of transition metals, group 13 or 14 elements, and carbon or nitrogen through selective removal of aluminum, therefore represent a class of electroactive nanomaterials whose biological behavior is intrinsically linked to their synthetic history [12]. Their physicochemical properties enable them to function not only as passive reinforcements but also as dynamic mediators of charge transfer and interfacial signaling within engineered tissues [35].

Table 2 provides a comparative overview of MXenes and other widely investigated two‐dimensional nanomaterials in the context of bioink engineering. Importantly, MXenes uniquely integrate high metallic conductivity with intrinsic hydrophilicity and tunable surface chemistry, enabling stable dispersion in aqueous bioink systems without extensive surfactant use. In contrast, graphene‐based systems often require oxidation or stabilizing agents to achieve comparable processability, while semiconducting nanomaterials such as MoS_2_ and black phosphorus are limited by either lower conductivity or environmental instability. This multifunctional convergence, conductivity, wettability, and chemical tunability, confers MXenes a distinct advantage for electroactive bioink development, particularly where simultaneous control over printability, electrical percolation, and biointerface compatibility is required.

**TABLE 2:** Comparison of MXenes with other 2D nanomaterials for bioink applications.

Property	MXenes (e.g., Ti_3_C_2_Tx)	Graphene/GO	MoS_2_	Black Phosphorus (BP)	Refs.
Electrical conductivity	Metallic conductivity (∼10^3^–10^5^ S m^−^^1^), tunable via surface terminations	High (graphene), reduced in GO due to defects	Moderate semiconducting behavior	Moderate, thickness‐dependent	[36, 37, 38, 39]
Hydrophilicity/dispersibility	Excellent aqueous dispersibility due to –OH, –O, –F surface terminations	Poor (graphene); improved in GO via oxygen groups	Limited dispersibility without modification	Poor; prone to rapid degradation in aqueous media	[40, 41, 42, 43]
Surface chemistry/functionalization	Rich, tunable surface chemistry enabling direct biofunctionalization	Requires chemical oxidation or post‐functionalization	Limited active surface chemistry	Reactive but unstable under ambient conditions	[44, 45, 46, 47]
Biocompatibility	Generally favorable; dependent on dose, oxidation state, and surface modification	Variable; GO may induce ROS‐mediated cytotoxicity	Moderate; limited long‐term studies	Limited by instability and degradation byproducts	[14, 48, 49, 50]
Printability in bioinks	Excellent colloidal stability and shear‐thinning behavior suitable for extrusion bioprinting	Requires modification for stable dispersion and rheology control	Limited printability due to aggregation	Poor due to instability and degradation	[51, 52, 53, 54]
Mechanical reinforcement	Strong reinforcement via 2D nanosheet network and interfacial interactions	High stiffness but poor dispersion limits uniform reinforcement	Moderate reinforcement capability	Limited due to instability	[43, 55, 56, 57]
Electrical–biological coupling	Strong coupling via conductive networks and surface charge interactions	Moderate; depends on percolation and dispersion	Limited	Limited	[58, 59, 60, 61]
Photothermal properties	Strong NIR absorption and efficient photothermal conversion	Moderate	Strong photothermal response	Strong but unstable under irradiation	[58, 62, 63, 64]
Degradation/stability	Oxidation‐sensitive but tunable via surface engineering	Highly stable	Chemically stable	Rapid degradation under oxygen/moisture	[65, 66, 67, 68]
Biomedical relevance	Tissue engineering, bioinks, biosensing, drug delivery	Drug delivery, scaffolds, coatings	Cancer therapy, sensing	Experimental regenerative applications	[52, 69, 70, 71]
Advantage for bioinks	Multifunctionality (conductivity + hydrophilicity + tunability)	High intrinsic conductivity	Photothermal capability	Biodegradability	[72, 73, 74, 75]
Primary limitation	Oxidation and long‐term biosafety concerns	Aggregation and poor dispersibility	Limited conductivity for bioelectronics	Environmental instability	[14, 49, 76, 77]

### 2.2 Biointerface Interactions and Stability

At the nano–bio interface, the biological performance of MXene nanosheets is governed not solely by intrinsic physicochemical properties but by dynamic transformations occurring in physiological environments. Upon exposure to biological fluids, MXenes rapidly adsorb proteins and biomacromolecules, forming a protein corona that defines their effective biological identity. The composition and stability of this corona are influenced by surface terminations, charge density, and nanosheet oxidation state, and may include both tightly bound “hard” corona components and more transient “soft” corona layers [78].

Protein corona formation can modulate cellular uptake pathways, immune recognition, and intracellular trafficking. For example, PEGylated MXene nanosheets generate distinct corona compositions compared to unmodified counterparts, influencing endocytic mechanisms and ROS dynamics in monocytic cells [79]. Corona‐mediated masking of reactive surface groups may alter integrin accessibility, modulate focal adhesion formation, and thereby influence downstream mechanotransduction signaling cascades. Such interfacial remodeling highlights that the biological identity of MXenes is not static but emergent, continuously redefined by environmental interactions.

The intrinsic hydrophilicity of MXene surfaces promotes interactions with extracellular matrix components and enhances uniform dispersion within hydrogel matrices, contributing to spatially consistent cellular microenvironments in bioprinted constructs. Furthermore, surface reactivity enables chemical conjugation with peptides, growth factors, or other bioactive ligands, facilitating tailored regulation of cell adhesion, proliferation, and lineage commitment.

Surface‐modified MXenes have demonstrated cell‐type‐dependent biological responses. Tunable surface chemistries influence integrin binding affinity and focal adhesion dynamics, key regulators of mechanotransduction pathways involved in stem cell differentiation and tissue maturation [17]. These effects are particularly relevant in electrically active tissues, where electroconductive substrates may synergistically modulate ion channel activity and cytoskeletal organization.

Surface terminations also impart redox‐active characteristics. While controlled redox interactions may stimulate pro‐regenerative pathways such as angiogenesis or osteogenic differentiation, excessive ROS generation, arising from surface reactions or oxidation‐derived TiO_2_ domains, can induce oxidative stress and cytotoxicity. Therefore, dose, exposure duration, and oxidation state collectively define a therapeutic window within which bioactivity is maximized and cytotoxicity is minimized.

Immune modulation represents an additional dimension of MXene–biointerface interactions. Emerging evidence suggests that surface chemistry may influence macrophage polarization profiles and cytokine secretion patterns, potentially biasing toward pro‐inflammatory or pro‐regenerative phenotypes depending on termination chemistry and surface modification [78]. Such immune responses are critical determinants of long‐term implant integration and functional tissue regeneration.

Surface modification strategies, including collagen functionalization and antioxidant polymer coatings, have been shown to reduce oxidative stress, stabilize colloidal behavior, and enhance cell viability through electrostatic and biomolecular interactions [80]. These approaches underscore the importance of interfacial engineering in balancing electroactivity with biosafety.

In summary, the layered morphology, heterogeneous surface terminations, and redox‐sensitive chemistry of MXenes render them highly adaptable nano–bio interface modulators. However, their biological performance emerges from a complex interplay between structure, surface chemistry, oxidative dynamics, and protein corona evolution. Precise control over these interfacial phenomena is therefore essential for the rational development of MXene‐based bioinks that achieve stable electroactivity, predictable cellular responses, and translational viability.

## 3 Engineering MXene Bioinks

The successful integration of MXenes into bioinks for 3D bioprinting requires a multi‐parameter optimization strategy in which formulation chemistry, nanosheet dispersion, cross‐linking kinetics, and rheological behavior are engineered concurrently. Unlike conventional hydrogel bioinks that primarily rely on polymer concentration and cross‐link density to define mechanical properties, MXene‐based systems must simultaneously balance electrical percolation, shear‐thinning behavior, network integrity, and cytocompatibility within a single composite platform. This creates a complex materials engineering landscape in which conductivity enhancement cannot compromise print fidelity or biological performance.

The central engineering challenge lies in achieving an electrically percolated nanosheet network at concentrations below cytotoxic thresholds while preserving extrusion stability and structural recovery post‐printing. Consequently, formulation design in MXene bioinks is increasingly guided by structure–rheology–function coupling principles rather than simple additive incorporation.

### 3.1 Formulation and Cross‐Linking Strategies

MXene incorporation typically involves uniform dispersion of Ti_3_C_2_T_x_ nanosheets within aqueous hydrogel matrices such as hyaluronic acid HA, alginate, GelMA, and composite polymer blends. Achieving homogeneous dispersion is critical, as nanosheet aggregation increases local stiffness heterogeneity, disrupts print resolution, and may generate localized oxidative stress. Early demonstrations showed that Ti_3_C_2_T_x_ nanosheets could be stably dispersed within HA/alginate systems, producing electroconductive inks capable of forming multilayered constructs with high shape fidelity while maintaining >95% cell viability in HEK‐293‐loaded scaffolds [11]. These findings established the feasibility of integrating conductive nanomaterials without compromising basic cytocompatibility.

Beyond feasibility, subsequent studies began leveraging MXene–polymer interfacial interactions to tune rheology and biological signaling. For example, silk fibroin methacrylate (SilMA) and pectin blends containing MXene exhibited enhanced shear‐thinning behavior and improved extrusion stability, supporting neural stem cell differentiation under electrical stimulation [14]. In such systems, hydrogen bonding and electrostatic interactions between polymer chains and MXene surface terminations contribute to dynamic physical cross‐linking, influencing viscosity, yield stress, and structural recovery after shear.

Importantly, MXene nanosheets may function not only as conductive fillers but also as active structural cross‐linking nodes. Their two‐dimensional morphology and high surface area enable multiscale stabilization mechanisms, including polymer chain adsorption, inter‐sheet stacking, and physical entanglement within hydrogel matrices. In composite systems incorporating complementary nanomaterials (e.g., graphene oxide), these interactions generate hierarchically reinforced porous architectures with improved stiffness and interconnected microchannels [81]. Such hybrid cross‐linking enhances post‐printing shape retention and mechanical robustness, critical parameters in extrusion‐based bioprinting where filament collapse and pore closure can compromise tissue maturation.

**FIGURE 1:**
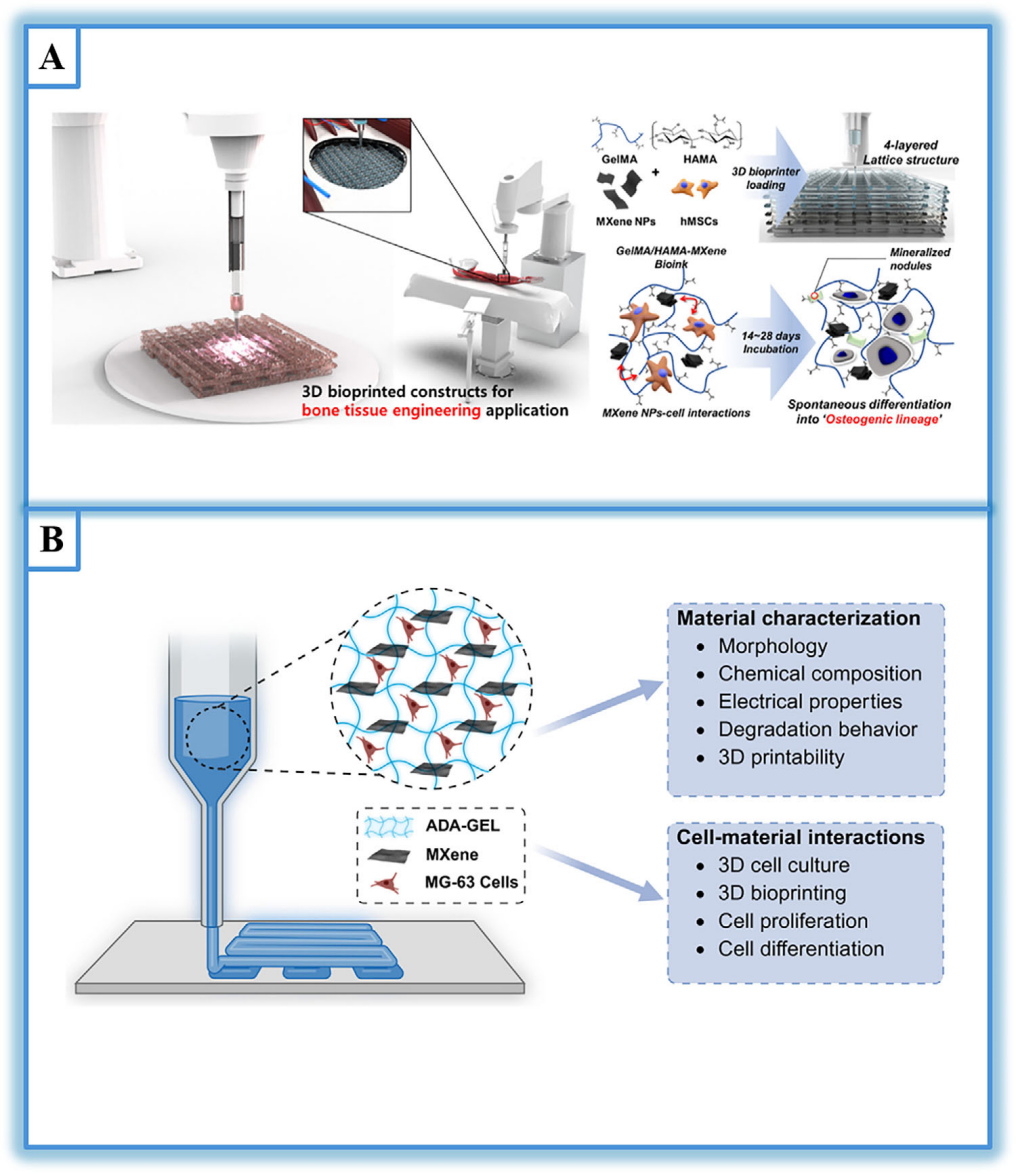
(A) Schematic abstract shows that GelMA/HAMA bioink containing MXene offers a promising biofunctional scaffold platform for bone tissue regeneration, supporting novel osteogenic differentiation of hMSCs in 3D printing. Reproduced with permission from [12]. Copyright 2023 Elsevier. (B) Schematic abstract demonstrates the tunable mechanical and electrical properties, 3D printability, and biocompatibility of ADA‐GEL/MXene nanocomposite hydrogels with MG‐63 cells. Reproduced with permission from [82]. Copyright 2025 Elsevier.

In methacrylated hydrogel systems such as GelMA and HAMA, MXene incorporation introduces additional complexity due to light absorption and charge transport behavior. Composite GelMA/HAMA–MXene bioinks exhibit improved rheological performance and mechanical integrity, enabling high‐resolution construct fabrication while supporting cell encapsulation. Notably, these systems have demonstrated spontaneous osteogenic differentiation of human mesenchymal stem cells without exogenous biochemical induction (Figure 1A) [12]. This behavior has been attributed to the synergistic interplay between MXene‐mediated electrical conductivity, altered microenvironment stiffness, and surface bioactivity, suggesting that electroconductive cues can partially substitute for soluble osteoinductive factors.

Similarly, Jo et al. reported that MXene‐incorporated GelMA/HAMA bioinks supported myogenic differentiation of C2C12 cells even in the absence of external differentiation media [83]. The electroactive microenvironment created by Ti_3_C_2_T_x_ nanosheets appears to modulate gene expression pathways associated with muscle maturation, potentially through enhanced ion flux and cytoskeletal organization. Importantly, in vivo volumetric muscle loss models demonstrated improved regeneration and attenuated inflammatory responses, indicating that formulation design influences not only in vitro differentiation but also host integration dynamics.

However, concentration‐dependent trade‐offs are consistently observed. Schöbel et al. demonstrated that MXene incorporation into alginate dialdehyde–gelatin (ADA–GEL) systems increases compressive modulus and conductivity in a dose‐dependent manner but may impair cell viability at higher loading levels (Figure 1B) [82]. This highlights a fundamental engineering constraint: increasing nanosheet concentration lowers percolation threshold and enhances electroactivity but may simultaneously interfere with cross‐linking efficiency, alter swelling kinetics, or elevate ROS generation.

Hybrid systems combining MXene with metallic nanoparticles further illustrate this balance. Boularaoui et al. showed that co‐integration of MXene and gold nanoparticles into GelMA matrices improves shear‐thinning behavior, electrical conductivity, and print resolution, facilitating enhanced myogenic maturation [8]. Nevertheless, excessive nanomaterial content can attenuate cross‐linking density and introduce cytotoxic risk, emphasizing the importance of defining a functional concentration window for each formulation [84].

Photocurable systems introduce additional engineering constraints. Lotfi et al. identified that MXene nanosheets exhibit strong UV–visible absorption, reducing light penetration depth and cross‐linking efficiency in GelMA‐based composites [85]. This optical attenuation imposes a thickness‐dependent curing limitation, particularly in high‐MXene formulations. Optimization of photoinitiator systems, including LAP, EY, and IG, has been shown to mitigate this effect, enabling tunable curing kinetics and improved structural integrity despite nanosheet incorporation. However, the interplay between MXene concentration, light attenuation, and cross‐linking rate defines a multidimensional design space that must be carefully navigated to preserve both electroactivity and print fidelity.

**FIGURE 2:**
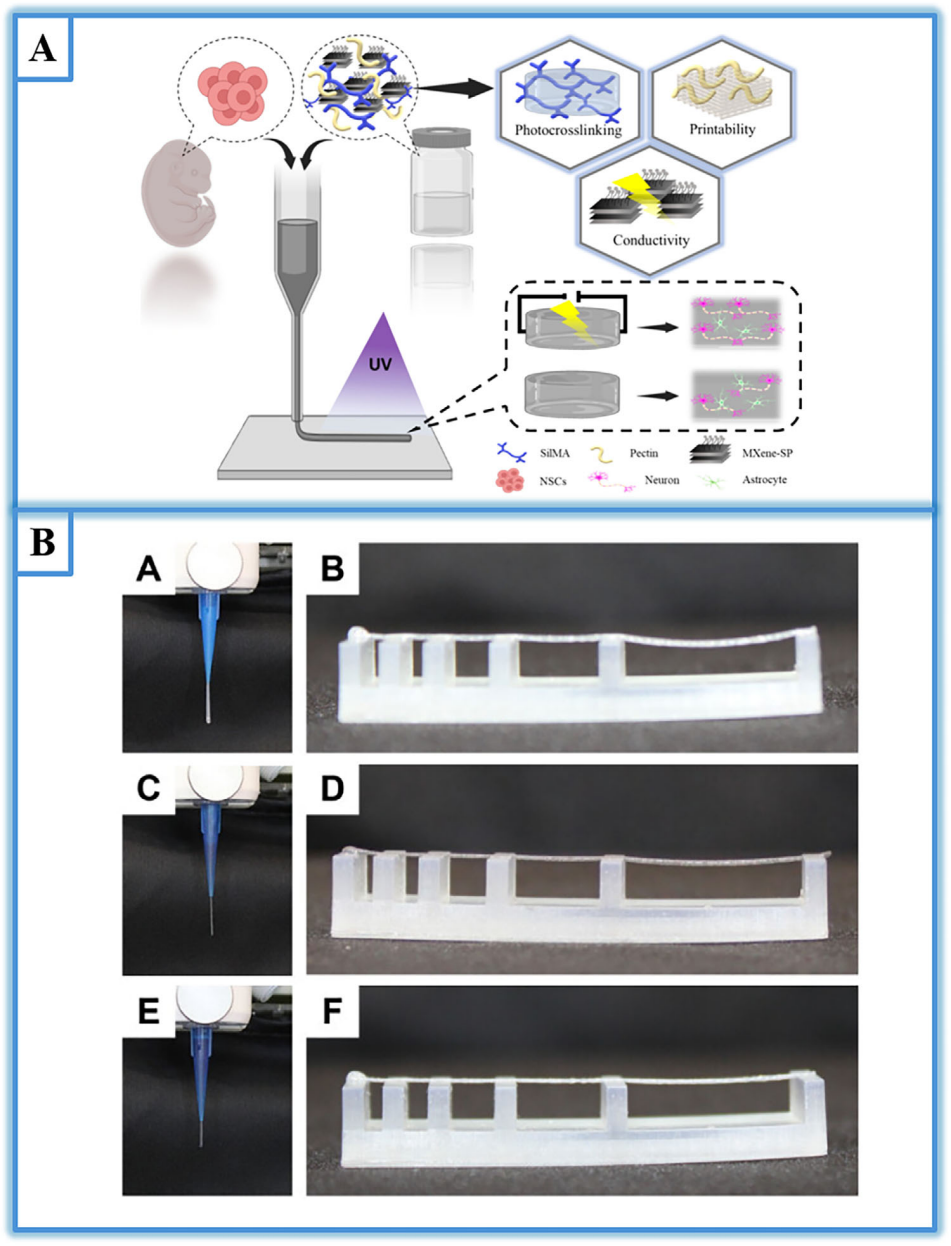
(A) Schematic representation of the conductive bioink formulation, its key physicochemical characteristics, and the 3D bioprinting of neural stem cell (NSC) spheroids under electrical stimulation. Reproduced with permission from [14]. Copyright 2025 American Chemical Society. (B) In the schematic summary, the printability of different bioinks was evaluated: (a,b) Suitable pressure and stability were determined for CELLINK ink using filament fiber and slump tests. (c,d) Printability and structural stability were analyzed in GGO ink. (e,f) Printing speed and stability were optimized in ink containing Ti_3_C_2_T_x_ MXene. Reproduced with permission from [58]. Copyright 2026 American Chemical Society.

Yeh et al. further demonstrated that MXene‐based hybrid bioinks incorporating SilMA and pectin can modulate mechanical stiffness and electrochemical properties in neural tissue engineering contexts (Figure 2A) [14]. Surface modification strategies enhancing dispersibility, such as phospholipid coatings, improve nanosheet homogeneity and prevent aggregation‐induced heterogeneity. Importantly, reductions in hydrogel stiffness combined with enhanced conductivity were associated with increased neural stem cell proliferation and synaptic activity under electrical stimulation, underscoring the coupling between material design and bioelectrical signaling.

Across these studies, formulation diversity highlights how polymer chemistry, nanomaterial concentration, and printing modality collectively define functional outcomes. As summarized in Table 3, MXene‐integrated bioinks span multiple hydrogel systems, extrusion platforms, and target tissues, yet converge on a common engineering principle: electrical percolation must be achieved within a rheologically stable and biologically permissive window. The comparative analysis underscores that MXene loading, cross‐linking strategy, and matrix selection directly modulate not only conductivity but also structural integrity, immune response, and lineage‐specific differentiation outcomes.

**TABLE 3:** Representative MXene‐integrated multimaterial bioinks and hybrid architectures for advanced 3D bioprinting applications.

Bioink composition	Bioprinting method	Distinctive properties	Target application	Refs.
GelMA, HAMA incorporated with Ti_3_C_2_T_x_ MXene	Extrusion‐based bioprinting (BioX, Cellink)	The hybrid hydrogel demonstrates high shape fidelity and mechanical stability after printing, with interconnected microporous architecture that supports cell viability and myogenic maturation. Additionally, the presence of MXene contributes to enhanced regenerative outcomes and mitigates adverse immune responses.	Skeletal muscle tissue engineering	[83]
GelMA, HAMA, and Ti_3_C_2_T_x_ MXene	Extrusion‐based bioprinting (BioX, Cellink)	MXene‐reinforced GelMA/HAMA scaffolds significantly enhance hMSC adhesion, proliferation, and long‐term survival, while inducing spontaneous osteogenic differentiation, highlighting their suitability for bone tissue regeneration strategies.	Bone regeneration	(Figure 1A) [12]
Alginate combined with Ti_3_C_2_T_x_ MXenes	Droplet‐based bioprinting (BioX, Cellink)	The incorporation of MXenes leads to a decline in cell viability accompanied by elevated reactive oxygen species generation; however, the system enables temperature‐responsive modulation, allowing controlled tumor suppression and model versatility.	In vitro cancer models	(Figure 6B) [86]
GelMA/β‐TCP/sodium alginate (Sr^2+^ ‐doped) with Ti_3_C_2_ MXene	Extrusion‐based bioprinting (Bio‐Architect SR)	This multifunctional bioink exhibits pronounced antibacterial activity and anti‐tumoral behavior, while simultaneously promoting osteogenic differentiation through synergistic interactions between MXene, β‐TCP, and Sr^2+^ ions.	Infected bone tissue repair	[87]
GelMA integrated with AuNPs and Ti_3_C_2_ MXene	Extrusion‐based bioprinting (Inkredible+, Cellink)	The combined inclusion of gold nanoparticles and MXene markedly improves electrical conductivity and print resolution, resulting in constructs with high cell viability and enhanced electrically mediated myogenic differentiation.	Muscle tissue constructs	(Figure 3A) [8]
Hyaluronic acid/alginate‐based hydrogel containing Ti_3_C_2_ MXene	Extrusion‐based bioprinting (Allevi 2, USA)	The MXene‐infused HA/Alg hydrogel displays favorable rheological behavior, elevated electrical conductivity, and excellent biocompatibility and biodegradability, rendering it suitable for neural tissue engineering applications.	Neural tissue engineering	[11]

Collectively, these studies reveal that MXene bioink performance emerges from a tightly coupled network of parameters: nanosheet concentration, surface chemistry, polymer matrix selection, cross‐linking strategy, and curing kinetics. Conductivity enhancement is inseparable from rheological modification, and mechanical reinforcement must be balanced against oxidative stability and cytocompatibility. Rather than functioning as passive additives, MXene nanosheets actively reshape hydrogel network architecture, microenvironment stiffness, electrical percolation behavior, and cellular mechanotransduction pathways.

Therefore, rational MXene bioink engineering demands a formulation framework in which electrical functionality, shear‐thinning behavior, and biological compatibility are co‐optimized within defined concentration and processing windows. This evolving paradigm marks a transition from empirical nanomaterial incorporation toward predictive, multifunctional bioink design tailored for electrically active tissue regeneration.

### 3.2 Rheology and Printability

Rheological behavior is a defining determinant of MXene‐based bioink performance, governing extrusion flow, filament formation, and post‐deposition structural integrity. In extrusion‐based bioprinting, optimal bioinks must exhibit shear‐thinning flow under high shear rates within the nozzle, followed by rapid viscosity recovery and elastic stabilization upon deposition. MXene‐based systems typically display non‐Newtonian power‐law behavior, where viscosity decreases with increasing shear rate, minimizing extrusion pressure while protecting encapsulated cells from excessive mechanical stress (Figure 2B) [58]. In addition to rheological characterization, qualitative extrusion‐based assessments such as filament string tests and filament collapse tests are frequently employed to evaluate printability. The filament string test assesses the ability of a bioink to extrude as a continuous and uniform filament without droplet formation, providing insight into optimal nozzle size and extrusion pressure. Complementarily, the filament collapse test evaluates shape retention and filament bridging capability across defined gaps, reflecting the balance between viscosity, elasticity, and printing speed. These simple yet informative methods are widely used to optimize extrusion parameters and ensure structural fidelity in MXene‐containing bioinks.

From a mechanistic standpoint, the rheological response of MXene bioinks emerges from nanosheet–polymer interactions and percolation dynamics. Moderate MXene concentrations increase zero‐shear viscosity and yield stress due to hydrogen bonding, electrostatic interactions, and physical entanglement between nanosheets and polymer chains. These interactions enhance filament continuity and strand stacking fidelity. However, beyond an optimal loading window, excessive nanosheet concentration increases flow resistance, elevates extrusion pressure, and may induce nozzle clogging or heterogeneity in filament diameter. Power‐law rheological models have been used to correlate MXene concentration with flow behavior indices and print resolution outcomes, revealing a nonlinear relationship between nanosheet loading and shear‐thinning exponent values [10].

Oscillatory rheology further clarifies this balance. A favorable post‐printing profile is characterized by storage modulus (G′) exceeding loss modulus (G″) after shear cessation, indicating elastic dominance and structural recovery. In MXene‐reinforced systems, the G′/G″ ratio is strongly influenced by nanosheet dispersion state and interfacial bonding strength. Polymers such as pectin and GelMA provide network elasticity, while MXene incorporation increases network connectivity, shifting the viscoelastic spectrum toward a more solid‐like response without compromising cell viability [14]. Importantly, rapid thixotropic recovery, defined as restoration of G′ after high‐shear deformation, is essential to prevent filament collapse during multilayer stacking.

In multicomponent hydrogel systems designed for bioelectronic skin applications, Ti_3_C_2_Tx incorporation into alginate/gelatin/methylcellulose matrices has demonstrated concentration‐dependent rheological modulation [88]. At low concentrations, MXene does not significantly perturb viscoelastic balance, preserving extrusion stability. Intermediate concentrations (∼1% w/v) improve filament uniformity and strand definition, suggesting improved yield stress and shear recovery kinetics. Conversely, excessive loading disrupts viscoelastic equilibrium, increasing apparent viscosity and compromising print precision. Notably, electrical conductivity enhancement persists across this range, highlighting the necessity of defining a concentration‐dependent design window that balances rheological performance with electroactivity.

Similar rheological behavior has been observed in HA/Alg–MXene systems, where homogeneous nanosheet dispersion produces stable shear‐thinning flow suitable for multilayer construct fabrication [11, 89]. In these systems, the introduction of MXene establishes conductive pathways without significantly altering extrusion pressure requirements, illustrating that optimized dispersion can decouple conductivity enhancement from excessive viscosity increase. This decoupling represents a key engineering objective in MXene bioink development.

Zhou et al. provided systematic rheological characterization of MXene dispersions, demonstrating that nanosheet suspensions inherently exhibit a dual viscoelastic character. Under applied shear, viscous behavior (G″) dominates, facilitating extrusion, while elastic behavior (G′ > G″) rapidly re‐emerges after shear cessation, enabling structural retention. Yield stress and plateau G′ values increase with nanosheet interaction density, reinforcing that dispersion homogeneity and inter‐sheet connectivity directly determine print stability. These findings position MXene dispersions as tunable rheological modifiers capable of supporting diverse biofabrication strategies [90].

Collectively, these studies demonstrate that rheological optimization in MXene‐based bioinks is governed by three coupled parameters: nanosheet concentration, dispersion homogeneity, and polymer network compatibility. Electrical percolation and rheological stabilization must be achieved within a narrow operational window, where extrusion pressure, filament fidelity, and cell viability remain balanced. The identification of this rheology–percolation design space is central to predictive MXene bioink engineering.

### 3.3 Functional Properties of MXene Bioinks

Beyond printability, MXene integration confers multifunctional capabilities that extend across mechanical reinforcement, electrical conductivity, biointerface modulation, and stimuli‐responsive behavior. These properties do not act independently; rather, they emerge from a multiscale interplay between nanosheet structure, hydrogel network architecture, and biological signaling [89].

Post‐printing cross‐linking transforms extruded filaments into mechanically stable constructs. In ionic systems such as alginate, Ca^2+^ ‐mediated gelation operates in parallel with MXene‐induced physical reinforcement, generating hybrid networks with improved compressive modulus and structural resilience [58]. In photocross‐linkable systems such as GelMA and SilMA, covalent polymer networks are reinforced by MXene nanosheets acting as stress‐transfer nodes. These nanosheets distribute mechanical load and improve cyclic stability, attributes particularly relevant for load‐bearing or contractile tissue [26].

**FIGURE 3:**
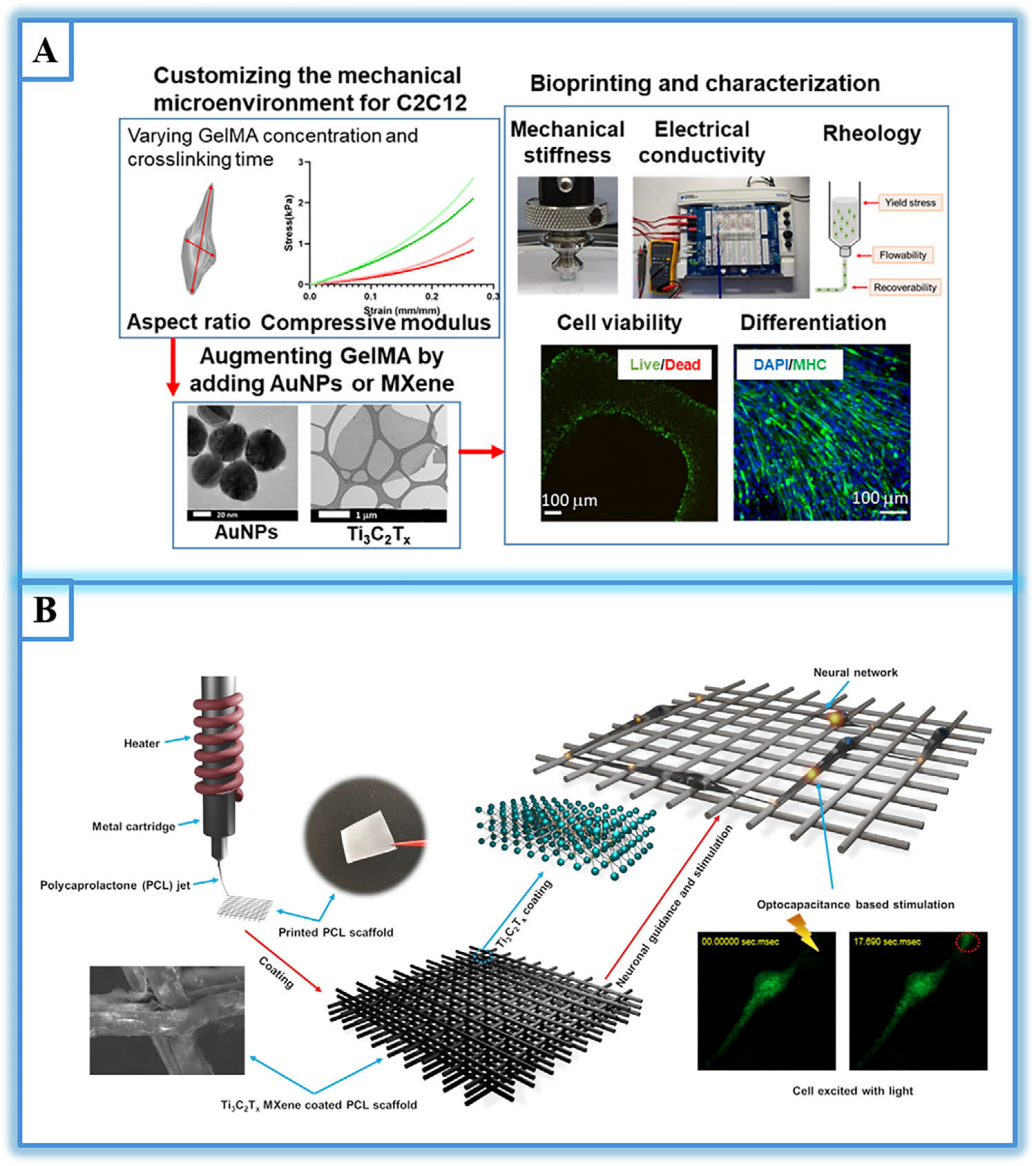
(A) Schematic summary shows that GelMA bioinks enhanced with AuNP and MXene support the viability, differentiation, and 3D tissue formation of C2C12 myoblasts by improving conductivity, rheological properties, and printability. Reproduced with permission from [8]. Copyright 2021 American Chemical Society. (B) Schematic abstract demonstrates the production of mechanically robust scaffolds via temperature‐controlled PCL printing and the provision of a cell‐friendly surface with Ti_3_C_2_T_x_ MXene coating to support optocapacitance‐based neural stimulation. Reproduced with permission from [91]. Copyright 2024 Nature.

Electrical functionality is a defining advantage of MXene incorporation. At relatively low filler concentrations, Ti_3_C_2_T_x_ nanosheets establish percolated conductive networks that significantly elevate hydrogel conductivity [58]. As previously reported, Boularaoui et al. reported conductivity values of 0.65 ± 0.04 and 0.94 ± 0.12 S m^−1^ for GelMA hydrogels containing 50 and 100 µg mL^−1^ MXene, respectively (Figure 3A) [8]. These values fall within physiologically relevant conductivity ranges for skeletal muscle and neural tissues, supporting electrically mediated cell communication and functional maturation.

Percolation modeling suggests that conductivity increases nonlinearly with MXene concentration until a threshold is reached, beyond which aggregation and heterogeneity may reduce effective transport pathways [10]. Therefore, defining the percolation threshold relative to cytotoxicity and cross‐linking efficiency is essential. Excessive loading may disrupt polymerization kinetics, alter swelling behavior, or induce ROS generation, establishing a conductivity–biocompatibility trade‐off that must be carefully managed [34].

MXenes also contribute bioactive interfacial properties. Their high specific surface area enables adsorption of serum proteins and biologically relevant ions. Jo et al. reported increases of 154% in serum protein adsorption, 176% in calcium ion adsorption, and 177% in potassium ion adsorption in MXene‐containing systems [83]. Given the central role of Ca^2+^ and K^+^ flux in excitation–contraction coupling, such ion‐interactive behavior reinforces the suitability of MXene‐based bioinks for excitable tissues.

In extrusion‐based systems, MXene addition has been shown to reduce effective shear stress experienced by encapsulated cells, creating a more protective printing environment [87]. For example, incorporation of 50 µg mL^−1^ MXene into 5 wt% GelMA significantly enhanced cell proliferation while maintaining structural stability [8]. These findings indicate that MXene nanosheets may simultaneously modulate rheological flow fields and post‐printing microenvironment stiffness, influencing both immediate cell survival and long‐term differentiation.

Beyond bulk incorporation, MXene surface functionalization strategies extend functionality to printed scaffold interfaces. Ti_3_C_2_T_x_‐coated polycaprolactone architectures fabricated via melt electrowriting demonstrate enhanced hydrophilicity and neuronal alignment (Figure 3B) [91]. Moreover, MXene's intrinsic photothermal response under near‐infrared (NIR) irradiation enables optically triggered modulation of cellular activity, expanding their utility toward bioelectronic and neuromodulatory applications.

Stimuli‐responsive behavior further broadens application scope. MXene‐mediated photothermal effects can induce localized heating, enabling controlled drug release or matrix stiffness modulation, contributing to emerging 4D bioprinting paradigms [10]. This dynamic functionality transforms MXene bioinks from static scaffolding materials into responsive bioelectronic platforms.

Beyond extrusion‐based bioinks, MXene‐integrated conductive hydrogels synthesized via one‐pot strategies have demonstrated enhanced mechanical robustness, self‐healing behavior, and fibroblast adhesion, highlighting the broader applicability of MXenes in epidermal bioelectronics and wound‐healing platforms [92].

Collectively, the functional performance of MXene‐based bioinks arises from a tightly coupled multiscale system in which nanosheet dispersion, network architecture, electrical percolation, and biointerface chemistry converge. Optimizing these parameters requires balancing electroactivity, mechanical reinforcement, and cytocompatibility within a defined formulation window. Thus, MXene‐enabled bioinks should be regarded not merely as conductive scaffolds but as dynamically engineered bioactive systems capable of modulating cellular signaling pathways and tissue‐level functionality [93].

## 4 Biological Performance

The biological performance of MXene‐enabled bioinks can be conceptualized through a multiscale structure–interface–cell–tissue continuum in which physicochemical properties are translated into functional cellular and tissue‐level outcomes. As summarized in Table 4, recent studies demonstrate that MXene nanosheets simultaneously modulate hydrogel mechanics, electrical conductivity, protein adsorption dynamics, and ionic interactions, thereby reshaping the local microenvironment experienced by encapsulated or seeded cells. These material‐level modifications propagate to the cellular scale, influencing mechanotransduction pathways, voltage‐sensitive ion channel activity, cytoskeletal organization, and lineage‐specific gene expression.

**TABLE 4:** Representative MXene‐based bioink systems for 3D bioprinting and their mechanistic contributions.

Application domain	Bioink composition/System	Fabrication strategy	Biological/Functional outcomes	Mechanistic insight	Translational relevance	Refs.
Bioink optimization	MXene‐integrated commercial bioinks	Extrusion‐based 3D bioprinting	High cell viability (>95%); tunable viscosity and print fidelity	MXene nanosheets modulate shear‐thinning behavior and percolation networks	Enables customizable bioink platforms adaptable to multiple tissues	(Figure 2B) [58]
Neural tissue engineering	SilMA/Pectin + MXene‐SP	3D bioprinting + electrical stimulation	Enhanced neuronal differentiation and neurite outgrowth	Conductive microenvironment enhances Ca^2+^ signaling and neurogenic pathways	Potential for neuroregenerative therapies and bioelectronic interfaces	[14]
Bone cancer dual therapy	MXene quantum dots + bioactive scaffold	3D printing	Simultaneous tumor ablation and bone regeneration	Photothermal conversion + ROS generation + osteoinductive signaling	Theranostic platforms for post‐resection bone repair	[94, 95]
Skeletal muscle regeneration	GelMA/HAMA + Ti_3_C_2_Tx MXene	Extrusion‐based bioprinting	Enhanced myogenesis without exogenous growth factors	Electrical conductivity modulates myogenic signaling and cell alignment	Reduces reliance on biochemical factors in muscle TE	[83]
Neural differentiation	MXene–Matrigel composite hydrogel	3D culture/printing	Increased NSC proliferation and differentiation	Electroconductive matrix supports neurogenic lineage commitment	Promising for CNS repair strategies	[99]
Bone tissue engineering	GelMA/HAMA + MXene (hMSC‐laden)	3D bioprinting	Spontaneous osteogenic differentiation without supplements	Surface charge + ion interaction mimic osteogenic microenvironment	Reduces need for osteoinductive factors	(Figure 1A) [12]
Hybrid composite scaffolds	rGO–MXene hydrogels	Scaffold fabrication	Enhanced cell adhesion, migration, and proliferation	Hybrid conductive networks improve electron transport and ECM interaction	Improved structural stability and multifunctionality	[96, 97]
Infected bone defects	MXene‐based hydrogel + NIR response	3D printed scaffold	Antibacterial activity + enhanced bone regeneration	Photothermal effect induces bacterial ablation + promotes osteogenesis	Infection‐resistant implants	[87]
Cardiac tissue engineering	MXene‐patterned PEG hydrogels	Aerosol jet printing	Improved electrical coupling and synchronized contraction	Anisotropic conductive pathways guide cardiomyocyte maturation	Bioelectronic cardiac patches	[98]

In electroresponsive tissues, including neural, muscular, cardiac, and skeletal systems, the formation of conductive percolation networks enhances cell–cell electrical coupling and promotes maturation processes that are traditionally dependent on exogenous biochemical cues. MXene surface chemistry further contributes to biological regulation by mediating ion adsorption and biointerface interactions, supporting osteogenic, myogenic, and neurogenic signaling cascades. Thus, MXene integration operates through coupled mechanical–electrical–interfacial mechanisms rather than through isolated conductivity enhancement.

Emerging in vivo evidence extends these findings beyond in vitro differentiation, indicating that MXene‐based constructs can facilitate tissue regeneration while modulating inflammatory responses. Multifunctional systems incorporating MXene quantum dots or NIR‐responsive hydrogels demonstrate additional therapeutic capabilities, including photothermal antibacterial and antitumoral effects [94, 95]. Hybrid architectures integrating MXene with rGO or spatial patterning strategies further illustrate how conductive networks and interfacial engineering can enhance cell adhesion, migration, and synchronized contraction in engineered cardiac tissues [58, 96, 97, 98].

Importantly, these biological advantages remain inherently linked to concentration‐dependent constraints, oxidative stability, and long‐term biosafety considerations. Excessive nanosheet loading may disrupt polymer networks, elevate oxidative stress, or compromise immune compatibility, underscoring the need for defined electroactivity–biocompatibility windows. Consequently, rigorous evaluation of biological performance must extend beyond cell viability metrics to include electrochemical stability, immune modulation, and degradation behavior under physiologically relevant conditions.

Together, these findings establish a unifying mechanistic paradigm in which MXene nanosheets function as multifunctional regulators of bioink‐mediated cellular environments. By concurrently modulating mechanical cues, electrical conductivity, and interfacial bioactivity, MXene‐based systems enable structurally robust and biologically instructive constructs, forming the basis for the in vitro, in vivo, and translational analyses that follow.

### 4.1 In Vitro Mechanisms

The in vitro biological performance of MXene‐based bioinks must be evaluated within a mechanistic framework that links physicochemical parameters to intracellular signaling cascades and functional maturation. Beyond conventional viability assays, emerging studies indicate that MXene incorporation reshapes the cellular microenvironment through coupled mechanical reinforcement, electrical conductivity, and interfacial charge interactions. These factors collectively regulate proliferation, cytoskeletal tension, ion‐channel activity, and lineage‐specific transcriptional programs.

#### 4.1.1 Cytocompatibility and Cell Proliferation

Cytocompatibility remains a foundational requirement for MXene‐enabled bioinks. Early demonstrations showed that Ti_3_C_2_Tx nanosheet incorporation into HA/Alg matrices maintained >95% viability in HEK‐293 cells [11], establishing that properly dispersed MXene does not induce acute cytotoxicity. However, cytocompatibility in MXene‐based systems extends beyond short‐term survival metrics and is strongly influenced by nanosheet concentration, oxidation state, and surface chemistry.

Mechanistically, several parameters govern cell compatibility. First, nanosheet dispersion influences local stiffness heterogeneity and shear stress distribution during printing, which in turn affects cytoskeletal organization and proliferation rates. Second, surface charge density modulates protein adsorption profiles and integrin clustering at the cell–material interface. Integrin‐mediated adhesion activates focal adhesion kinase (FAK) signaling and downstream pathways that regulate cell spreading and proliferation. Thus, MXene surface chemistry indirectly contributes to proliferation through modulation of adhesion‐dependent mechanotransduction.

In SilMA/pectin/MXene systems, neural stem cells exhibited enhanced proliferation under electrical stimulation [14]. This suggests that electroconductive matrices facilitate voltage‐sensitive ion channel activation and Ca^2+^ influx, which are known regulators of cell cycle progression. Similarly, GelMA/HAMA‐MXene composites supported high hMSC viability and spontaneous osteogenic differentiation [12], indicating that MXene‐mediated microenvironmental cues extend beyond simple cytocompatibility to active regulation of stem cell behavior.

Surface engineering further refines cytocompatibility. Poly‐L‐lysine (PLL) modification of Ti_3_C_2_ nanosheets alters zeta potential and electrostatic interactions with cell membranes [100, 101]. This modification improves colloidal stability and reduces cytotoxic effects within defined concentration ranges, highlighting that cytocompatibility is not intrinsic to MXene but emerges from controlled surface chemistry.

Importantly, concentration‐dependent effects remain critical. Excessive MXene loading may increase reROS) production through surface redox activity or oxidation‐derived TiO_2_ domains, potentially inducing oxidative stress and mitochondrial dysfunction. Therefore, a cytocompatible window must balance electrical percolation and oxidative stability. Current literature predominantly reports short‐term assays, and systematic evaluation of long‐term proliferation, genomic stability, and immune signaling remains limited.

Collectively, in vitro cytocompatibility in MXene‐based bioinks depends on a convergence of dispersion quality, surface termination chemistry, concentration thresholds, and matrix compatibility. Rational bioink design must therefore define safe electroactive concentration windows rather than relying solely on high viability percentages.

#### 4.1.2 Lineage‐Specific Differentiation and Functional Outcomes

Beyond supporting proliferation, MXene‐based bioinks actively influence lineage specification through electromechanical and interfacial mechanisms. Differentiation outcomes in these systems appear to arise from the interplay between matrix stiffness, conductive network formation, and ion‐mediated signaling pathways.

In neural applications, SilMA/pectin/MXene bioinks enhanced neuronal marker expression and synaptic activity under electrical stimulation [14]. These outcomes likely involve Ca^2+^‐dependent signaling cascades, where MXene‐mediated conductivity amplifies externally applied electrical cues, facilitating membrane depolarization and downstream transcriptional activation associated with neurogenesis. Enhanced neurite extension suggests improved cytoskeletal remodeling and actin dynamics, processes regulated by integrin–FAK and Rho‐family signaling pathways.

In musculoskeletal models, MXene‐infused GelMA/HAMA matrices promoted myogenic differentiation of C2C12 cells [83]. The combined effects of mechanical reinforcement and electrical conductivity likely influence mechanosensitive pathways such as YAP/TAZ nuclear translocation and cytoskeletal tension regulation. Electrical coupling between adjacent cells may further enhance synchronized differentiation through gap junction–mediated communication.

**FIGURE 4:**
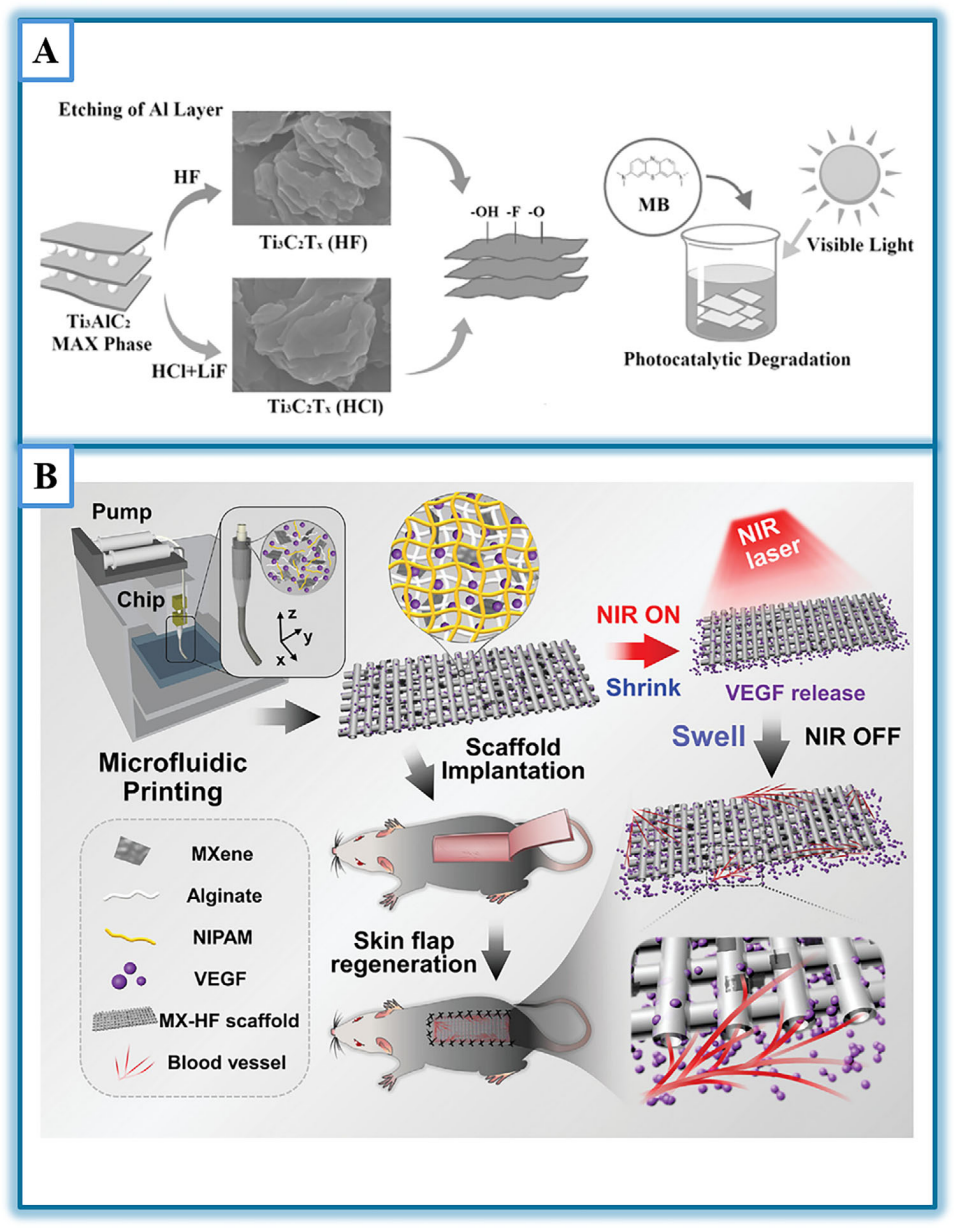
(A) Schematic summary, the structural and optical properties of Ti_3_C_2_T_x_ MXene synthesized by HF and LiF/HCl methods were compared; MXene produced by HF showed higher photocatalytic activity and methylene blue decomposition efficiency. Reproduced with permission from [102]. Copyright 2025 Springer Nature. (B) Schematic representation of microfluidic 3D‐printed MX‐HF scaffolds designed for skin flap regeneration. Fabricated through a coaxial capillary microfluidic approach, the scaffolds exhibit NIR‐triggered photothermal shrinkage and swelling, promoting cell and tissue infiltration within the channels. Furthermore, the regulated release of VEGF from the dynamic scaffold architecture enhances neovascularization and supports the regeneration of large skin flap defects. Reproduced with permission from [103]. Copyright 2022 Wiley.

Cardiac tissue engineering provides a compelling demonstration of spatially controlled electroactivity. Aerosol jet–printed Ti_3_C_2_Tx patterns on PEG hydrogels guided cardiomyocyte alignment and increased expression of cardiac‐specific markers including MYH7, SERCA2, and TNNT2 (Figure 4A) [102]. Enhanced conduction velocity and synchronous contraction indicate improved electrophysiological integration. These findings suggest that MXene patterning enables anisotropic electrical guidance, mimicking native myocardial architecture through coordinated structural and conductive cues.

In bone tissue contexts, spontaneous osteogenic differentiation observed in MXene‐containing bioinks [11, 12] may arise from surface charge–mediated ion interactions and modulation of extracellular Ca^2+^ availability. Adsorption of biologically relevant ions can influence osteogenic transcription factors and mineralization pathways, reinforcing the role of MXene as an electrochemical niche modulator.

Despite these promising outcomes, mechanistic decoupling remains incomplete. The relative contributions of conductivity, stiffness modulation, and surface chemistry to lineage commitment are not fully resolved. Furthermore, most findings are derived from short‐term in vitro models, and translation to sustained in vivo functional integration remains insufficiently explored.

Thus, MXene‐mediated differentiation should be interpreted as an emergent property of electromechanical coupling rather than as a purely conductive effect. Future studies integrating transcriptomic profiling, ion‐channel analysis, and mechanotransduction pathway mapping will be essential to fully elucidate the molecular basis of lineage specification in MXene‐enabled systems.

#### 4.1.3 Mechanotransduction and Electrical Signaling Pathways

The influence of MXene‐based bioinks on cellular behavior extends beyond structural support, encompassing the coordinated regulation of mechanotransductive and electrochemical signaling pathways. Conductive bioinks modulate cell fate not only through scaffold stiffness and topographical cues but also by reshaping local electrical microenvironments that interface with intrinsic cellular signaling networks.

At the electromechanical interface, MXene‐mediated conductivity enhances charge transport within the hydrogel matrix, facilitating membrane depolarization and Ca^2+^ influx through voltage‐sensitive ion channels. Calcium signaling acts as a central integrator of electrical cues, activating downstream cascades including MAPK/ERK and PI3K–Akt pathways, which regulate proliferation, differentiation, and survival [10]. Although direct pathway dissection within MXene systems remains limited, related studies in conductive hydrogel platforms demonstrate enhanced calcium flux and integrin‐mediated signaling under electrical stimulation, mechanisms likely underpinning the differentiation outcomes observed in MXene bioinks.

Simultaneously, MXene surface terminations and protein corona formation influence integrin clustering and focal adhesion dynamics at the cell–material interface. Integrin engagement activates focal adhesion kinase (FAK), which transduces mechanical and biochemical cues into cytoskeletal reorganization and gene expression programs. Through modulation of local stiffness and surface charge density, MXene incorporation may alter FAK signaling and downstream mechanosensitive regulators such as YAP/TAZ, thereby linking matrix mechanics to lineage specification [104].

Mechanical stiffness represents a critical regulator of mechanotransduction. Stem cells interpret substrate rigidity through cytoskeletal tension and focal adhesion maturation. Softer matrices (0.1–1 kPa) promote neurogenic differentiation, intermediate stiffness (5–50 kPa) favors myogenesis, and rigid substrates (>100 kPa) induce osteogenic pathways [96]. Conventional bioinks often fall outside optimal stiffness windows, with collagen systems being overly compliant (0.2–0.8 kPa) and agarose systems excessively rigid (168–230 kPa) [97]. MXene incorporation enables simultaneous reinforcement and conductivity enhancement, allowing tuning of hydrogel moduli within physiologically relevant ranges while preserving electroactivity [11, 58].

In skeletal muscle engineering, conductive 3D scaffolds facilitate synchronized electrical propagation, enhancing myotube alignment and maturation [105, 106, 107]. MXene‐enabled bioinks combine mechanical reinforcement with electrical signal transmission, creating an electromechanically active niche that more closely recapitulates native muscle microenvironments. This dual modulation supports coordinated mechanotransduction and ion‐channel activation, processes essential for functional tissue formation.

Cardiac tissue engineering further illustrates the integration of structural and electrophysiological cues. Aerosol jet–printed Ti_3_C_2_Tx MXene patterns on PEG hydrogels generate anisotropic conductive pathways that guide cardiomyocyte alignment and maturation [108, 109]. Upregulation of cardiac markers such as MYH7, SERCA2, and TNNT2, along with increased synchronous contraction and improved conduction velocity, indicates that spatially controlled MXene deposition enhances both structural organization and electrophysiological integration. These findings suggest that MXene‐based biointerfaces can replicate aspects of native myocardial anisotropy through coordinated mechanical and electrical guidance [98].

Beyond externally applied stimulation, MXene‐based platforms have also been engineered to harness endogenous bioelectrical signals. In maxillofacial and soft tissue repair models, Ti_3_C_2_Tx nanosheets capture intrinsic wound‐site electrical gradients and convert them into localized microcurrents, establishing self‐regulating electroactive microenvironments [110, 111, 112]. Biological evaluations demonstrate enhanced collagen deposition, vascularization, and re‐epithelialization comparable to externally applied electrical stimulation [113, 114]. Such systems highlight the capacity of MXene to function as a self‐powered biointerface that dynamically couples physiological electrical signals to regenerative processes.

In neural repair contexts, multifunctional MXene hydrogels incorporating stromal cell‐derived factor‐1α (SDF‐1α) have demonstrated activation of PI3K–Akt and calcium‐dependent pathways, accompanied by reduced oxidative stress and macrophage polarization toward a regenerative phenotype [34]. These findings underscore that MXene‐mediated electrical environments intersect with immunomodulatory signaling, linking electroactivity to microenvironmental regulation [115].

At the material level, electronic structure and defect chemistry further influence biointerface behavior. Ti_3_C_2_Tx ‐coated polycaprolactone scaffolds exhibit enhanced hydrophilicity and vacancy‐rich surface structures that affect charge transport and interfacial interactions [116, 117]. Surface defects and termination heterogeneity may modulate electron density distribution and protein adsorption patterns, thereby shaping cell attachment and proliferation dynamics [118, 119].

Collectively, MXene‐based bioinks modulate mechanotransduction and electrical signaling through a coupled network of stiffness tuning, conductive percolation, ion‐channel activation, and integrin‐mediated adhesion. Rather than acting as passive conductive fillers, MXene nanosheets establish electromechanically integrated microenvironments that regulate intracellular signaling cascades across neural, muscular, cardiac, and soft tissue systems. However, mechanistic decoupling of conductivity‐driven versus stiffness‐driven effects remains incomplete, and systematic pathway‐level investigations are required to fully elucidate MXene‐mediated signal transduction [120, 121].

#### 4.1.4 Cellular Responses in 3D Contexts

Understanding cellular responses within 3D microenvironments is critical for accurately evaluating the biological performance of MXene‐based bioinks, as 3D systems more faithfully replicate native tissue architecture, spatial mechanical gradients, and mass transport conditions. While 2D studies provide initial insights into cytocompatibility and signaling, they fail to capture the complex interplay between cell–matrix interactions, nutrient diffusion, and electromechanical coupling that defines functional tissue formation.

In 3D constructs, MXene integration influences not only cell viability but also spatial organization, network formation, and long‐term functional maturation. Several reports demonstrate that MXene‐augmented scaffolds maintain high viability (>95%) after extended culture while supporting neurite extension, myotube alignment, and multicellular network formation [83]. These outcomes suggest that conductive percolation networks established within volumetric matrices enable distributed electrical signaling rather than localized surface effects typical of 2D systems.

Matrix porosity and microarchitecture, which can be modulated by MXene concentration and polymer composition, play a decisive role in 3D cellular behavior. MXene nanosheets may alter cross‐linking density and pore size distribution, thereby influencing oxygen diffusion, nutrient transport, and metabolic waste clearance. In thick constructs, diffusion limitations can generate hypoxic gradients that modulate stem cell fate and angiogenic signaling. Therefore, tuning porosity alongside conductivity is essential to prevent necrotic core formation and to sustain long‐term cell function. Reported correlations between scaffold porosity, proliferation rates, and differentiation outcomes highlight that architectural parameters are inseparable from bioelectrical effects in 3D biofabrication.

Importantly, electrical signaling behaves differently in 3D matrices. In volumetric hydrogels, conductive nanosheet networks establish interconnected pathways that distribute electric fields across the construct, potentially influencing cell–cell communication and synchronization over larger distances. This spatial propagation of electrical cues is particularly relevant in excitable tissues such as neural and muscular systems, where coordinated signaling governs functional maturation.

Wang et al. developed a microfluidic‐assisted, MXene‐integrated hollow fibrous scaffold exhibiting dynamic responsiveness to NIR stimulation (Figure 4B) [103]. The system demonstrated reversible structural modulation, controlled shrinkage and expansion, which enhanced cellular infiltration within the 3D architecture. In addition, stimulus‐responsive release of vascular endothelial growth factor (VEGF) significantly promoted endothelial cell proliferation, migration, and angiogenic activity. In vivo skin flap models confirmed improved neovascularization, reduced inflammatory response, and decreased apoptosis, illustrating how MXene‐enabled structural adaptability can couple mass transport enhancement with regenerative signaling.

Such findings emphasize that MXene‐based systems in 3D contexts operate through a combined framework of electromechanical modulation and microenvironmental regulation. Conductive networks influence ion flux and cell synchronization, while dynamic scaffold architectures regulate infiltration and vascularization. Together, these properties facilitate tissue‐level integration beyond isolated cellular effects.

Nevertheless, mechanistic understanding of how 3D‐distributed electric fields generated by MXene networks influence intracellular signaling remains incomplete. Most current studies rely on phenotypic assessments rather than pathway‐level mapping within volumetric constructs. Future investigations integrating spatial transcriptomics, electrophysiological mapping, and real‐time ion flux analysis will be essential to fully elucidate the biological consequences of MXene‐mediated electroactivity in 3D microenvironments.

### 4.2 In Vivo Evidence and Biosafety

While in vitro investigations provide mechanistic insight into cellular behavior, the translational viability of MXene‐based bioinks ultimately depends on their in vivo performance. Preclinical models are essential to evaluate biocompatibility, host–material interactions, immune modulation, degradation behavior, vascular integration, and functional tissue restoration. These parameters collectively determine whether electroactive biofabricated constructs can transition from experimental platforms to clinically relevant regenerative therapies.

Importantly, in vivo environments introduce complex variables absent in controlled in vitro systems, including dynamic immune responses, vascularization requirements, mechanical loading, and systemic clearance pathways. Therefore, successful translation of MXene‐integrated bioinks requires not only regenerative efficacy but also controlled inflammatory responses, predictable degradation kinetics, and long‐term biosafety.

#### 4.2.1 Muscle Regeneration Models

Among emerging applications, skeletal muscle regeneration models provide some of the most compelling in vivo evidence supporting MXene‐based bioinks. Volumetric muscle loss (VML) represents a clinically challenging condition characterized by irreversible tissue loss and limited intrinsic regenerative capacity. In murine VML models, extrusion‐bioprinted hydrogels composed of GelMA and HAMA incorporating Ti_3_C_2_Tx MXene nanoparticles demonstrated significantly enhanced muscle regeneration compared with polymer‐only controls [58].

Histological analyses revealed increased expression of both early (e.g., MyoD) and late (e.g., MyHC) myogenic markers, along with improved myofiber organization and reduced fibrotic tissue formation. These findings indicate that MXene integration supports structural regeneration while promoting functional maturation of muscle fibers. Importantly, functional assessments demonstrated improved contractile performance and muscle strength recovery in treated animals, suggesting that MXene‐mediated conductivity contributes to coordinated myofiber development and neuromuscular integration.

Beyond structural regeneration, immunomodulatory effects appear to contribute significantly to the observed outcomes. MXene‐containing constructs were associated with attenuated inflammatory infiltration and reduced expression of pro‐inflammatory markers relative to controls. Such findings suggest that MXene integration may influence macrophage polarization dynamics, potentially shifting the microenvironment toward a regenerative phenotype. The conductive and surface‐reactive properties of Ti_3_C_2_Tx nanosheets may modulate protein adsorption and cytokine signaling, thereby influencing host–material interactions during the acute inflammatory phase.

Mechanistically, the regenerative benefits likely arise from combined electromechanical coupling within the scaffold microenvironment. MXene incorporation enhances hydrogel mechanical stability and viscoelastic behavior, maintaining structural integrity under physiological loading. Simultaneously, conductive percolation networks may facilitate synchronized electrical signaling between regenerating myofibers, supporting myoblast fusion and alignment. However, these benefits are strongly concentration‐dependent. Optimized MXene loadings promote proliferation and myogenic differentiation, whereas excessive concentrations can induce cytotoxic effects, possibly due to strong electrostatic interactions with cellular membranes and localized oxidative stress [83, 122].

Matrix stiffness further modulates regenerative outcomes. Moderately cross‐linked GelMA/HAMA constructs provide a permissive mechanical niche that supports cellular infiltration and differentiation, whereas excessively stiff matrices impair proliferation and functional integration. Thus, muscle regeneration outcomes reflect a delicate balance between scaffold mechanics, electrical conductivity, and host immune response.

Collectively, these in vivo findings indicate that MXene‐mediated electromechanical modulation plays a central role in coordinating structural regeneration with functional recovery in skeletal muscle tissue. Nevertheless, long‐term biosafety data remain limited, particularly regarding degradation products, systemic biodistribution, and chronic immune responses. Comprehensive longitudinal studies will be essential to validate the safety profile of MXene‐integrated constructs before clinical translation.

#### 4.2.2 Bone and Hard Tissue Regeneration

Extending MXene‐based bioinks from soft tissues to load‐bearing skeletal environments introduces additional biomechanical and biological constraints, including high mechanical demand, mineralization requirements, vascularization coupling, and immune compatibility. Recent in vivo studies increasingly demonstrate that MXene‐integrated scaffolds can support robust osteogenesis while simultaneously enabling multifunctional therapeutic interventions. In critical‐sized bone defect models, composite scaffolds combining MXenes with bioceramics or polymeric matrices have shown enhanced osteoblastic activity and accelerated new bone formation without detectable cytotoxicity at optimized concentrations. MXene‐modified ceramic scaffolds significantly upregulated bone‐related proteins and promoted mineralized matrix deposition, suggesting that conductive nanosheet incorporation can potentiate osteogenic signaling cascades in vivo.

Photoactivated Ti_3_C_2_Tx MXene systems further reveal multi‐modal regenerative mechanisms. Under controlled irradiation, MXene presentation upregulated pro‐angiogenic factors and osteogenic differentiation markers both in vitro and in vivo, indicating that electroactive and photothermal properties may synergistically enhance vascularized bone regeneration [123]. These findings suggest that MXene incorporation supports not only structural mineralization but also angiogenesis‐coupled osteogenesis, which is critical for defect healing.

Beyond conventional regeneration, MXene‐based scaffolds are increasingly engineered to integrate oncological therapy with skeletal repair. This theragenerative paradigm is particularly relevant following malignant bone tumor resection, where residual tumor cells and compromised osseointegration represent major clinical challenges.

**FIGURE 5:**
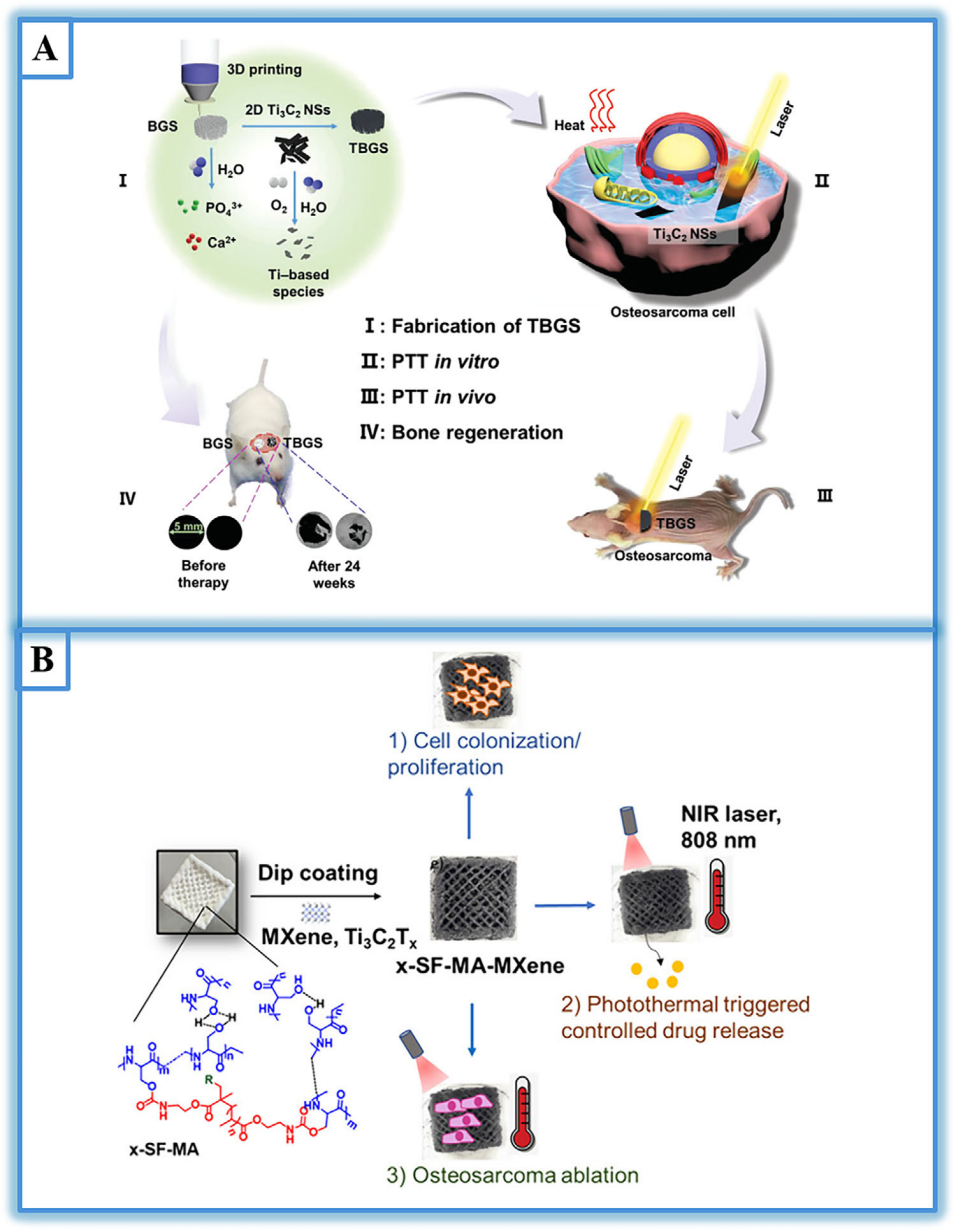
(A) Schematic diagram illustrating the preparation of multifunctional TBGS scaffolds designed for both photothermal treatment of bone tumors and bone defect repair. The illustration includes: (I) the fabrication process of TBGS scaffolds; (II) in vitro photothermal destruction of osteosarcoma cells; and (III) bone tissue regeneration and in vivo photonic therapy of osteosarcoma following TBGS implantation and subsequent NIR irradiation. Reproduced with permission from [95]. Copyright 2019 Wiley. (B) Schematic summary shows that MXene‐reinforced 3D‐printed double‐cross‐linked silk fibroin aerogel scaffolds provide osteosarcoma treatment through NIR‐induced photothermal effect and drug delivery, while promoting bone regeneration. Reproduced with permission from [124]. Copyright 2023 American Chemical Society.

Silk fibroin (SF)‐based aerogel scaffolds incorporating Ti_3_C_2_ MXene nanosheets exemplify this dual‐function approach (Figure 5B) [124]. Through 3D printing and photo‐cross‐linking, these constructs achieve controlled architecture and mechanical stability while leveraging strong NIR photothermal conversion for localized tumor ablation. Importantly, these scaffolds simultaneously support preosteoblastic proliferation and mineral deposition, demonstrating that tumoricidal and osteogenic functions can coexist within a single platform.

More advanced multifunctional systems integrate two‐dimensional Nb_2_C MXene with S‐nitrosothiol‐functionalized mesoporous silica and 3D‐printed bioactive glass frameworks [125]. In these hybrid constructs, NIR‐II–activated photothermal effects enable efficient tumor cell eradication, while nitric oxide (NO) release provides antibacterial and microenvironment‐modulating activity. Concurrent calcium and phosphate ion release from the bioactive glass matrix further promotes osteogenesis. The coordinated sequence of tumor ablation, angiogenesis induction, and bone regeneration reflects a sophisticated theranostic strategy.

Similarly, Ti_3_C_2_ MXene integrated within 3D‐printed bioactive glass (BG) scaffolds achieved complete tumor regression in xenograft models through localized photothermal effects while simultaneously enhancing new bone formation at defect sites (Figure 5A) [95]. These studies collectively highlight that MXene‐based scaffolds can bridge oncological therapy and regenerative reconstruction within a unified system. Infection‐related bone loss introduces an additional layer of complexity, as effective treatment requires not only structural bone repair but also rapid bacterial elimination, inflammatory control, and restoration of an osteogenic microenvironment. To address these competing demands, multifunctional MXene‐containing bioink/scaffold systems have been developed to integrate photothermal antibacterial activity with osteoinductive and mechanically supportive features.

A representative example is a hybrid GelMA/β‐tricalcium phosphate (β‐TCP)/Sr^2+^ ‐cross‐linked alginate system incorporating Ti_3_C_2_ MXene, which demonstrated effective photothermal bactericidal activity under NIR irradiation against both Gram‐positive and Gram‐negative bacterial strains [87]. In this design, Ti_3_C_2_ MXene primarily contributed to NIR‐responsive photothermal antibacterial performance, whereas β‐TCP and Sr^2+^ provided osteoconductive and osteoinductive cues that enhanced the osteogenic differentiation of bone marrow‐derived mesenchymal stem cells. In vivo implantation in infected mandibular defect models further showed accelerated infection resolution and improved bone regeneration, illustrating how MXene‐based composite scaffolds can couple infection control with tissue reconstruction. Importantly, this example also demonstrates that the therapeutic value of MXene‐containing bioinks depends on the coordinated design of multiple functional components rather than on conductivity or photothermal conversion alone.

Combinatorial systems incorporating berberine (BBR) within 3D‐printed biphasic calcium phosphate (BCP) scaffolds further extend this strategy [126]. MXene‐mediated photothermal activation enabled stimulus‐responsive BBR release, enhancing antibacterial efficacy while preserving scaffold mechanical integrity. These results demonstrate the feasibility of integrating drug delivery, infection control, and structural support within a single regenerative platform.

Beyond functional outcomes, recent investigations have begun elucidating the molecular mechanisms underlying MXene‐driven bone regeneration. In Ti_2_AlN MXene–polycaprolactone (PCL) composite scaffolds, incorporation of 5% MXene significantly improved surface wettability, cell adhesion, and early‐stage osteogenic differentiation, as indicated by increased alkaline phosphatase (ALP) activity [127]. In vivo studies in rat tibial and rabbit maxillofacial defect models confirmed enhanced bone formation.

Transcriptomic analyses revealed activation of Wnt/β‐catenin signaling pathways and calcium‐binding protein regulation, suggesting that MXene‐mediated electrochemical microenvironments may influence canonical osteogenic cascades. These findings indicate that MXene integration not only reinforces scaffold mechanics but also modulates intracellular signaling networks critical for bone regeneration.

Collectively, these studies illustrate a transition from single‐function osteogenic scaffolds toward multifunctional MXene‐enabled platforms capable of integrating structural support, electroactivity, photothermal therapy, antimicrobial control, and biochemical signaling. Such systems exemplify an advanced theragenerative paradigm in bone tissue engineering.

Nevertheless, several translational challenges remain. Long‐term degradation kinetics, systemic biodistribution of MXene degradation products, surface chemistry variability, and fabrication standardization require rigorous investigation. Inconsistencies in nanosheet termination chemistry and concentration‐dependent cytotoxicity further complicate cross‐study comparisons. Addressing these issues will be critical for advancing MXene‐based hard tissue scaffolds from promising preclinical models toward safe and standardized clinical implementation.

#### 4.2.3 Immunogenicity and Biocompatibility

Biocompatibility represents a decisive parameter in the translational assessment of MXene‐based bioinks, as regenerative efficacy must be coupled with controlled immune responses and predictable biosafety profiles. Preclinical studies consistently report minimal adverse immune reactions and acceptable host tolerance when MXene nanosheets are appropriately integrated within composite hydrogels. In skeletal muscle regeneration models, MXene‐augmented scaffolds exhibited reduced inflammatory infiltrates and a shift toward pro‐regenerative macrophage phenotypes compared with polymer‐only controls [10]. These findings suggest that MXene incorporation may modulate the early inflammatory phase and influence the transition from acute inflammation toward constructive tissue remodeling. Macrophage polarization dynamics appear to be particularly relevant. Several in vivo observations indicate enhanced expression of markers associated with M2‐like phenotypes alongside reduced pro‐inflammatory cytokine production. Given the critical role of macrophage‐mediated cytokine gradients in orchestrating angiogenesis and matrix deposition, this immunomodulatory effect may contribute significantly to the regenerative outcomes observed in muscle and bone models. However, the precise molecular mechanisms governing MXene‐induced immune modulation remain insufficiently defined.

Importantly, MXene biocompatibility is not intrinsic but highly dependent on physicochemical parameters. Surface termination groups (─F, ─OH, ─O), lateral nanosheet dimensions, oxidation state, and synthesis routes collectively determine cellular interactions and toxicity profiles. At nanoscale dimensions (1–100 nm), MXene particles can be internalized via endocytosis, influencing intracellular trafficking and potential cytotoxic responses [19, 128]. Dose‐dependent effects are consistently observed: optimized concentrations maintain high viability, whereas excessive nanosheet loading can compromise membrane integrity through electrostatic interactions with negatively charged phospholipid bilayers.

Oxidative stress emerges as a central determinant of MXene‐associated cytotoxicity. Residual oxidizing species generated during synthesis, along with redox‐active surface terminations, may elevate intracellular reactive oxygen species (ROS) levels, triggering apoptosis‐related pathways [129]. While moderate ROS levels can support regenerative signaling, uncontrolled oxidative stress may induce mitochondrial dysfunction and inflammatory cascades. This dual role underscores the importance of defining concentration windows that balance bioactivity and safety.

In vivo clearance studies indicate partial elimination of MXenes through renal and fecal pathways within short timeframes; however, long‐term biodistribution, degradation kinetics, and potential accumulation in reticuloendothelial organs remain incompletely characterized. These gaps are particularly relevant for repeated or large‐volume implantations, where chronic exposure scenarios may arise.

Surface engineering strategies have demonstrated considerable promise in mitigating cytotoxicity and improving stability. Functionalization with biocompatible polymers, including polyethylene glycol (PEG), poly(lactic‐co‐glycolic acid) (PLGA), chitosan, poly‐L‐lysine, collagen, and silk fibroin, enhances colloidal stability, reduces nonspecific protein adsorption, and attenuates oxidative stress responses [26]. Emerging machine learning approaches further aim to predict cytotoxic outcomes based on synthesis parameters and surface chemistry, offering a data‐driven pathway for rational MXene optimization.

Collectively, current evidence indicates that the biosafety of MXene‐based systems is context‐dependent and governed by nanosheet chemistry, dose, and microenvironmental interactions. Rigorous standardization of synthesis protocols, longitudinal biodistribution studies, and immune profiling under clinically relevant conditions will be essential to ensure safe clinical translation [114].

#### 4.2.4 Functional Integration and Phenotypic Outcomes

Functional integration represents the ultimate benchmark for regenerative biomaterials, as successful tissue repair requires not only structural restoration but also recovery of physiological performance and long‐term host compatibility. MXene‐based biointerfaces offer a unique opportunity to couple electroactivity, mechanical reinforcement, and biointerface tunability to support integrated tissue remodeling.

In vivo evidence suggests that MXene‐containing scaffolds extend beyond passive support to actively modulate phenotypic outcomes. In skeletal muscle and bone models, improved alignment, matrix deposition, and enhanced tissue organization indicate that conductive scaffolds facilitate coordinated cellular behavior. Electrical conductivity may promote synchronized cell–cell communication, while surface charge interactions influence adhesion and cytoskeletal organization, collectively contributing to functional maturation.

**FIGURE 6:**
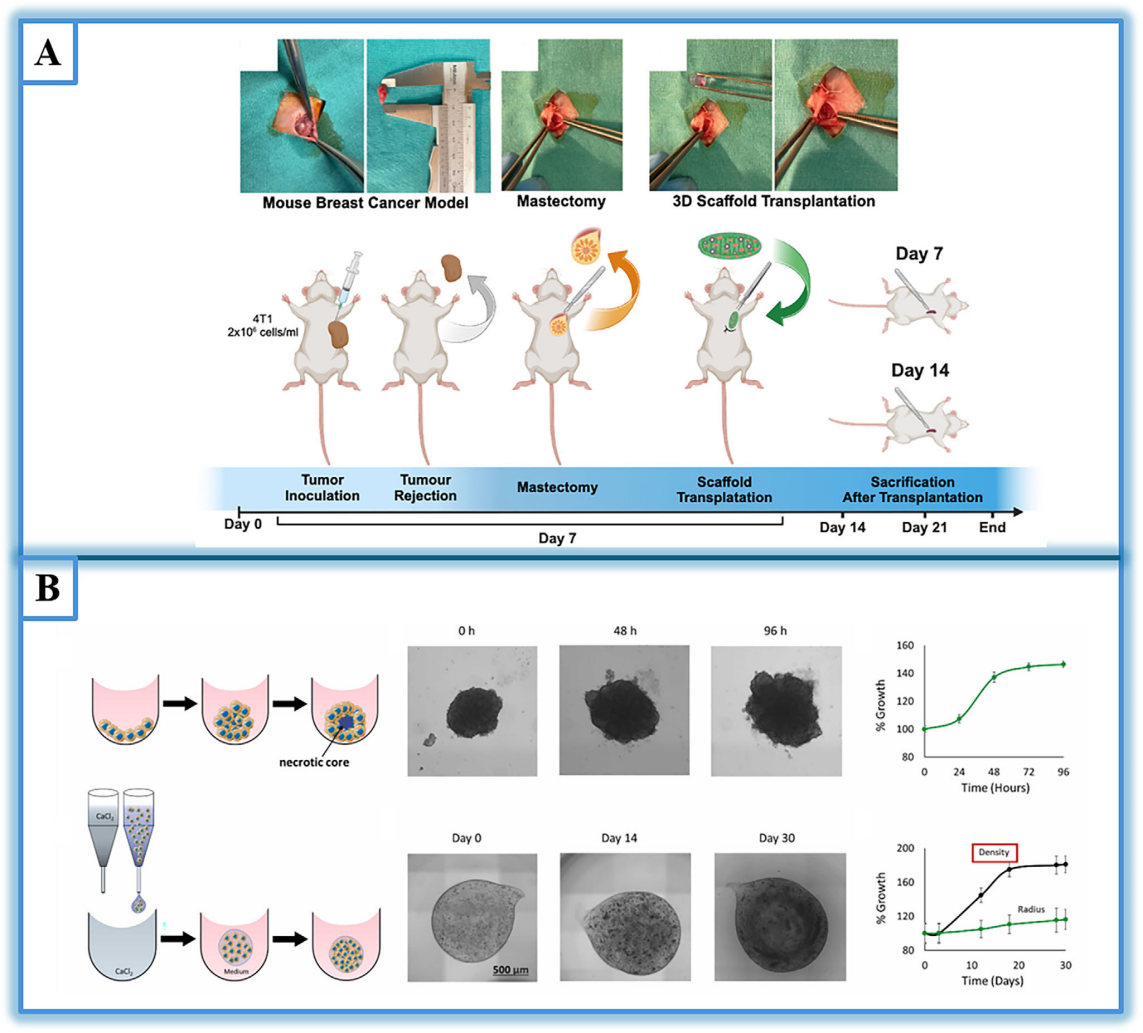
(A) Schematic summary shows breast cancer modeling, mastectomy, and implantation of 3D‐printed scaffolds. Tumor images and size measurements one week after breast tumor induction. Mastectomy procedure following tumor excision. Implantation of a 3D‐printed scaffold after mastectomy. Schematic representation of the in vivo experimental workflow. Reproduced with permission from [130]. Copyright 2025 Wiley. (B) Schematic summary shows the time‐dependent growth of 4T1 spheroids. Schematic representation of the formation of conventional spheroids using a 96‐well ultra‐low adhesion (ULA) plate. Development of conventional spheroids 0, 48, and 96 h after centrifugation; cells were seeded onto ULA plates and centrifuged at 300 g for 3 min. Increase in spheroid radius over time for conventionally prepared spheroids. Bioprinting procedure for spheroid production. Representative images of 4T1 spheroids produced by bioprinting on days 0, 14, and 30. Temporal changes in radius and density of bioprinted spheroids. Reproduced with permission from [86]. Copyright 2022 Elsevier.

Alkaya et al. demonstrated a 3D hydrogel scaffold incorporating MXene quantum dots (MQDs) and stem cells for simultaneous tissue regeneration and tumor recurrence prevention following breast cancer resection (Figure 6A) [130]. In murine models, MQD‐containing constructs suppressed early tumor recurrence while simultaneously promoting tissue remodeling, as confirmed by histological and immunohistochemical analyses. The dual‐function capability highlights how MXene‐based systems can integrate oncological control with regenerative reconstruction within a single microenvironment.

Beyond oncological contexts, functional integration in electroresponsive tissues such as cardiac and neural systems depends on restoring electrophysiological continuity. Conductive MXene networks may facilitate synchronized electrical propagation across engineered tissues, reducing conduction heterogeneity and supporting maturation of excitable cell populations. Although long‐term electrophysiological mapping remains limited, early data suggest improved contraction synchrony and organized tissue architecture in conductive constructs.

Nevertheless, long‐term integration dynamics remain underexplored. Questions persist regarding scaffold degradation rates relative to tissue remodeling, stability of conductive networks over time, and potential immune responses under chronic loading conditions. Moreover, the durability of electromechanical coupling after partial MXene degradation requires further investigation to determine whether functional benefits are transient or sustained.

In addition to regenerative applications, MXene‐mediated photothermal platforms have demonstrated functional tumor modulation in bioprinted 3D breast cancer spheroid models, where localized hyperthermia and ROS generation significantly reduced tumor viability and influenced immune cell migration patterns, underscoring the capacity of MXene‐enabled systems to actively regulate complex 3D microenvironments (Figure 6B) [86].

Overall, MXene‐based biomaterials demonstrate the capacity to orchestrate structural repair, phenotypic modulation, and therapeutic functionality within complex in vivo environments. However, achieving durable and clinically reliable integration will require comprehensive long‐term studies addressing degradation behavior, immune equilibrium, and electrophysiological stability under physiologically relevant conditions.

### 4.3 Mechanistic Framework of MXene‐Mediated Bioactivity in 3D‐Bioprinted Constructs

The preceding subsections document a rapidly expanding body of evidence showing that MXene‐containing bioinks support cytocompatible encapsulation, accelerate lineage‐specific differentiation, and enhance the functional maturation of 3D‐bioprinted constructs. Yet a recurring interpretive ambiguity runs through this literature: the observed biological effect is rarely attributed to a single, experimentally isolated MXene property. Instead, MXene loading simultaneously modulates electrical conductivity, viscoelastic/mechanical stiffness, surface termination chemistry, and the local ionic micro‐environment, all of which can converge on overlapping downstream signaling pathways (e.g., FAK/ERK, YAP/TAZ, Piezo1, connexin‐43, L‐type Ca^2+^ channels) [131, 132].

#### 4.3.1 Electrical Conductivity and Charge‐Transfer Phenomena

MXenes of the Ti_3_C_2_T_x_ family exhibit metallic‐to‐semiconducting electronic transport, with sheet resistance tunable over several orders of magnitude by composition (Ti_3_C_2_, Ti_2_C, Nb_4_C_3_, Mo_2_C), interflake contact engineering, oxidation state, and polymer‐matrix percolation threshold. In electroactive tissues, cardiomyocyte networks, skeletal myotubes, neural spheroids, and bone (where piezoelectric and streaming potentials operate), MXene scaffolds have been reported to (a) redistribute exogenous electrical stimulation more uniformly across the cell‐adherent interface, (b) accelerate the propagation of action‐potential‐like Ca^2+^ transients, (c) upregulate voltage‐gated ion channels and connexin‐43, and (d) amplify Piezo1‐mediated mechanosensitive currents. For example, in 3D‐bioprinted cardiac constructs, conductive additives such as MXenes have been benchmarked against reduced graphene oxide, carbon nanotubes, and gold nanorods in alginate–gelatin and GelMA–AlgMA hydrogels, where MXene loading produced conductivities on the order of ∼10^7^ S/m while preserving cell viability >90% by day 7 [133]. In skeletal muscle tissue engineering, low‐concentration GelMA bioinks containing 0.05 mg/mL Ti_3_C_2_T_x_ nanosheets exhibited conductivity of 0.65 ± 0.04 S/m (vs. ∼0.3 S/m for pure GelMA) and a 5–15% increase in myosin heavy chain expression in C2C12 myoblasts even in the absence of exogenous electrical stimulation [8, 134]. In neural applications, NSCs cultured on Ti_3_C_2_T_x_ films formed more active and synchronous network activity than on TCPS controls, displayed significantly longer axons (41.75 ± 2.75 µm vs. 29.84 ± 2.19 µm), and exhibited increased spontaneous excitatory postsynaptic current frequency; coupling with electrical stimulation further amplified NSC proliferation [135]. However, the literature largely attributes these outcomes to “conductivity” without measuring the actual transduced potential at the cell–material interface, or without designing matched‐conductivity controls that also match stiffness and surface chemistry. This conflation is the central concern raised in the present review and motivates the framework proposed here.

#### 4.3.2 Mechanical and Topographical Signaling

Increasing MXene content in a bioink almost invariably raises the storage modulus (G′), the equilibrium Young's modulus, or both, and frequently introduces sub‐micron topographical cues at flake edges that re‐model integrin clustering and focal‐adhesion maturation. These cues are translated into downstream YAP/TAZ nuclear translocation, MRTF‐A–SRF signaling, and ERK1/2 phosphorylation, which together govern lineage decisions in MSCs, myoblasts, and neural progenitor cells, consistent with the broader matrix‐mechanotransduction paradigm in which substrate stiffness directs stem‐cell fate through FAK/SRC/integrin and Hippo‐pathway effectors [136]. In MXene bioinks specifically, mechanical reinforcement is well documented: incorporation of 2 mg/mL Ti_3_C_2_T_x_ into alginate‐cellulose bioinks raised the compressive Young's modulus by ∼72% (to ∼0.39 S/m conductivity) [58]; in MXene/SilMA bioinks, increasing the MXene content raised the compressive modulus and the shear‐thinning index required for extrudable printability [14]; and in oxidized alginate‐gelatin MXene hydrogels, Young's modulus increased from 40 to 68 kPa upon MXene reinforcement [82]. Importantly, however, the few available studies that explicitly matched modulus across MXene‐loaded and MXene‐free bioinks (e.g., by adding compensatory inert filler) suggest that mechanical reinforcement alone can account for a substantial fraction of the observed osteogenic and myogenic benefit. This is consistent with the broader hydrogel literature in which stiffness, independently of conductivity, modulates lineage commitment.

#### 4.3.3 Surface Chemistry and Termination‐Group Effects

MXene flakes carry a heterogeneous mixture of ─OH, ─O, and ─F terminations whose ratios depend on the etchant (HF, LiF+HCl, mixed HF/HCl, halogen‐free NaOH), delamination route, intercalant, and post‐treatment annealing [26, 137, 138]. These terminations regulate (a) surface energy and water contact angle, (b) the composition and conformation of the adsorbed protein corona (vitronectin, fibronectin, albumin), and (c) the availability of hydrogen‐bond donors/acceptors at the biointerface. Halogen‐free NaOH‐etched Ti_3_C_2_T_x_ shows a water contact angle of ∼34.5° (vs. ∼49.8° for HF‐etched Ti_3_C_2_T_x_), an ∼3.6 eV work function, and preserved biocompatibility at concentrations up to 2000 µg/mL at which conventional fluorinated Ti_3_C_2_T_x_ caused ∼50% viability loss [139, 140]. Conversely, fluorinated MXene flakes release F^−^ ions upon partial oxidation that can promote ROS generation, disrupt mitochondrial membrane potential, and trigger pro‐inflammatory cytokine release, providing a mechanistic link between surface termination chemistry and inflammatory tone. At the cell‐interface level, the predominantly ─O/─OH‐terminated Ti_3_C_2_T_x_ has been shown to cause negligible hemolysis (0.8% at 200 µg/mL) in red blood cells and to preserve HUVEC morphology, whereas graphene oxide at the same concentration caused 50.8% hemolysis, an effect attributed largely to the distinct hydrophilic surface chemistry of MXene [141].

#### 4.3.4 Ion Adsorption, Intercalation, and Release Behavior

MXenes are well‐known cation intercalation hosts and reversibly accommodate Na^+^, K^+^, Ca^2+^, and Mg^2+^ between their galleries, with ion‐transport kinetics governed by interlayer hydration, defect density, and termination chemistry [142, 143]. In physiological media, partial oxidative degradation also liberates low concentrations of titanium species. The biological implications are dual‐edged: on the beneficial side, local Ca^2+^ enrichment at the cell interface may amplify L‐type Ca^2+^ channel activity and promote osteogenic mineralization or cardiogenic excitation–contraction coupling, independently of any electrical effect; on the detrimental side, Ti release above a poorly defined threshold can elevate intracellular ROS, compromise mitochondrial membrane potential, and trigger pro‐inflammatory cytokine release [139]. The available data suggest that the beneficial/detrimental crossover lies in the low‐µg/mL range and depends strongly on the oxidation state of the released species, but few studies have quantified release kinetics in 3D‐printed constructs under physiologically relevant perfusion. This mechanism is particularly relevant for MXene composites co‐loaded with bioactive ions: in Ti_3_C_2_T_x_ ‐decorated 3D‐printed ZnSr/β‐TCP ceramic scaffolds, for example, photothermal activation by Ti_3_C_2_ was combined with the gradual release of Ca^2+^, Zn^2+^, Sr^2+^, and PO_4_^3−^ ions to coordinate ROS scavenging, macrophage polarization from M1 to M2, and osteogenesis [144]; and in MXene/copper‐functionalized GelMA/Alg‐DA hydrogels, Ti_3_C_2_T_x_ nanosheets adsorbed free Cu^2+^ electrostatically to prevent explosive release while supporting osteogenic and angiogenic differentiation [145].

#### 4.3.5 Combinatorial and Synergistic Effects

Taken together, the four mechanisms above are not independent variables: conductivity is itself a function of interflake contact (which is dictated by stiffness and dispersion quality); surface chemistry is itself a function of oxidation (which releases ions); and ion adsorption is itself a function of interlayer hydration (which depends on polymer‐matrix interactions). Consequently, biological outcomes almost always reflect a convolution of these factors. We therefore propose that future MXene bioink studies adopt a multidimensional characterization matrix that simultaneously reports: (1) sheet resistance or bulk conductivity at the printing‐relevant solids content; (2) G′ and tan δ at the cell‐seeding frequency; (3) XPS‐derived ─OH/─O/─F ratios and Ti 2p oxidation state; and (4) Ca^2+^, Mg^2+^, and Ti release kinetics over 1, 7, and 14 days. Studies that cannot provide all four should refrain from attributing outcomes to any single mechanism [146, 147].

Table 5 summarizes the four primary mechanism classes alongside the MXene property they map to, the documented biological outcome, a representative bioink system, and the principal limitation of the currently available evidence.

**TABLE 5:** Mechanistic categorization of MXene bioink bioactivity.

Mechanism category	MXene property driving the effect	Biological outcome(s)	Bioink/system	Quantitative evidence	Limitation	Refs.
Electrical conductivity	High electronic conductivity; tunable sheet resistance; percolation‐dependent bulk conductivity	Cardiomyocyte Ca^2+^ handling, Cx43 upregulation, neuronal firing, osteogenic Piezo1 activation, myotube contractility	Ti_3_C_2_T_x_ ‐GelMA, Ti_3_C_2_T_x_ ‐alginate/gelatin, Ti_3_C–collagen, Ti_3_C_2_T_x_ ‐PEG	↑ beating rate, ↑ ALP under stimulation, ↑ MEA spike frequency; conductivities ∼0.65–∼10^7^ S/m depending on loading	Rarely decoupled from stiffness; transduced potential at cell interface rarely measured	[133, 148]
Mechanical/topographical	↑ G′, ↑ E, nanoscale flake‐edge roughness, anisotropic stress transfer	YAP/TAZ nuclear translocation, focal adhesion maturation, myogenic and osteogenic lineage commitment	MXene–alginate‐cellulose, MXene–SilMA, MXene–GelMA, MXene–HAMA	Modulus‐dependent myotube width; compressive modulus up to ∼470 kPa; Young's modulus raised from 40 → 68 kPa in alginate‐gelatin	Topography rarely quantified independently of MXene loading; few matched‐modulus controls	[8, 136, 149]
Surface chemistry/termination	–OH/–O/–F ratio, surface energy, protein‐corona composition, oxidation state (TiO_2_‐like vs. Ti–C)	Initial adhesion, inflammatory cytokine profile, ROS response, long‐term phenotypic stability	NaOH‐etched vs. HF‐etched Ti_3_C_2_T_x_; aged vs. fresh MXene; Ti_3_C_1.5_N_0.5_ carbonitride	↓ TNF‐α, ↑ vitronectin adsorption; contact angle 34.5° vs. 49.8°; hemolysis 0.8% vs. 50.8% (GO control)	Termination ratios rarely quantified; aging effects underreported	[26, 137, 139]
Ion adsorption/release	Ca^2+^, Mg^2+^, Na^+^, K^+^ intercalation; low‐level Ti release; partial oxidative degradation	L‐type Ca^2+^ activation, osteogenic mineralization, mitochondrial stress at high Ti	MXene–hydroxyapatite, MXene–ZnSr/β‐TCP, MXene/Cu–GelMA/Alg‐DA	Dose‐dependent ALP, ARS; Ti release kinetics tied to oxidation state	Threshold between beneficial and cytotoxic Ti release unclear; release kinetics in 3D‐printed constructs rarely measured	[142, 150]
Combinatorial/synergistic	Coupled variation of ≥2 of the above	Functional tissue‐level maturation in 3D‐bioprinted cardiac, skeletal muscle, bone, and neural constructs	Multi‐component MXene bioinks under combined electrical + mechanical stimulation; photothermal MXene composites	Tissue‐level functional readouts (contractile force, electrophysiology, BV/TV from micro‐CT)	Cannot be reduced to single‐property attribution; statistics typically underpowered for multivariate regression	[144, 146, 147]

## 5 Translational and Regulatory Considerations

The clinical translation of MXene‐based bioinks extends far beyond proof‐of‐concept demonstration and requires alignment with manufacturing scalability, regulatory classification, long‐term biosafety, and ethical deployment frameworks. While preclinical data demonstrate promising regenerative and multifunctional performance, the path toward clinical adoption demands rigorous standardization, risk stratification, and compliance with regulatory agencies such as the U.S. Food and Drug Administration (FDA) and the European Medicines Agency (EMA).

MXene‐enabled systems frequently fall into complex regulatory categories. Depending on formulation and intended use, they may be classified as medical devices, combination products (device–drug or device–biologic hybrids), or advanced therapy medicinal products (ATMPs). For example, photothermal tumor‐ablation scaffolds incorporating drug release or nitric oxide donors may require dual regulatory evaluation, significantly increasing translational complexity. Consequently, early‐stage design must consider regulatory classification to streamline approval pathways and reduce downstream barriers.

Unlike traditional polymer‐based biomaterials, MXene‐containing systems introduce electroactive nanocomponents whose degradation products, long‐term persistence, and biodistribution must be thoroughly characterized. Therefore, translational advancement requires not only demonstration of regenerative efficacy but also comprehensive toxicological profiling, chronic implantation studies, and validated degradation models.

### 5.1 Standardization, Synthesis, and Manufacturing Scalability

Reproducible and scalable synthesis constitutes a foundational requirement for regulatory acceptance. MXene fabrication commonly involves selective etching of MAX phases followed by delamination and surface modification. However, variations in etching chemistry, such as hydrofluoric acid–based methods versus minimally intensive layer delamination approaches, produce substantial differences in surface termination profiles (−F, −OH, −O), lateral nanosheet dimensions, oxidation kinetics, and colloidal stability [151].

These physicochemical variations directly influence rheological behavior, print fidelity, degradation rate, and biointerface interactions. Consequently, inconsistencies in synthesis protocols hinder the establishment of robust structure–property–function relationships and complicate interlaboratory reproducibility. From a regulatory standpoint, such variability poses significant obstacles to compliance with Good Manufacturing Practice (GMP) standards and quality control requirements mandated by agencies including the FDA and EMA.

Manufacturing scale‐up introduces additional technical challenges. Large‐scale production must ensure controlled delamination efficiency, narrow lateral size distribution, uniform surface chemistry, and long‐term dispersion stability. Even minor variations in nanosheet aggregation state can alter bioink viscosity, percolation thresholds, and electrical conductivity, thereby affecting biological performance.

To address these challenges, translational pipelines must incorporate standardized characterization frameworks, including surface chemistry quantification (e.g., XPS‐based termination analysis), nanosheet size distribution mapping, oxidation state monitoring, and accelerated aging studies. Integration of in‐line quality control systems during manufacturing will be critical to achieve batch‐to‐batch consistency.

Furthermore, regulatory approval will require validated sterilization protocols compatible with MXene stability. Conventional sterilization methods, such as gamma irradiation, autoclaving, or ethylene oxide treatment, may alter surface terminations or accelerate oxidation, potentially affecting functionality and safety. Thus, sterilization compatibility studies must be incorporated early into manufacturing design.

Ultimately, establishing scalable, quality‐controlled synthesis–manufacturing pipelines capable of meeting GMP standards will be essential for translating MXene‐based bioinks from laboratory innovation to clinical‐grade biomaterials [152].

### 5.2 Sterilization and Biological Stability

The clinical deployment of MXene‐based bioinks necessitates sterilization strategies that preserve both physicochemical integrity and functional bioactivity. Unlike conventional polymeric biomaterials, MXene‐containing constructs incorporate electroactive nanosheets whose surface terminations and oxidation state critically determine conductivity, dispersibility, and biointerface interactions. Consequently, sterilization‐induced alterations may significantly affect performance.

Standard sterilization modalities, including autoclaving, gamma irradiation, and ethylene oxide exposure, pose distinct risks. Thermal sterilization may accelerate MXene oxidation and structural fragmentation. Gamma irradiation can induce surface defect formation and modify termination chemistry, potentially altering electrical conductivity and redox behavior. Ethylene oxide, while milder, may leave residual contaminants or modify surface functional groups. Such changes may influence protein adsorption, integrin engagement, and cell signaling pathways.

Despite these risks, systematic investigations examining sterilization‐induced alterations in MXene systems remain scarce, representing a major translational gap. Establishing validated sterilization protocols tailored to MXene stability is therefore essential. Potential strategies include low‐temperature plasma sterilization, filtration‐based approaches for bioink precursors, or aseptic manufacturing pipelines that minimize post‐fabrication exposure.

Equally critical is the long‐term biological stability of MXene constructs under physiological conditions. Ti_3_C_2_T_x_ and related MXenes are prone to oxidation in aqueous, oxygen‐rich environments, leading to gradual loss of conductivity and alteration of surface chemistry. Degradation may result in nanosheet fragmentation and formation of titanium oxide species, which can influence both scaffold mechanics and cellular responses.

From a translational safety perspective, such instability raises concerns regarding degradation byproducts, persistence in reticuloendothelial organs, and long‐term accumulation. Although preliminary studies indicate partial renal and fecal clearance, comprehensive pharmacokinetic and biodistribution analyses remain limited. Chronic implantation models and accelerated aging studies under simulated physiological conditions will be necessary to define acceptable degradation windows.

To enable regulatory progression, standardized testing frameworks must be developed to quantify sterilization effects, oxidation kinetics, conductivity retention, and degradation behavior in biologically relevant environments. Defining performance thresholds, rather than relying solely on qualitative observations, will be critical for establishing predictable safety profiles and facilitating regulatory approval [10].

### 5.3 Biocompatibility, Immunomodulation, and Biosafety Evaluation

Comprehensive biosafety evaluation of MXene‐based systems must extend beyond acute cytocompatibility to encompass long‐term immune modulation, hemocompatibility, genotoxicity, systemic biodistribution, and degradation product profiling. Given the nanoscale and electroactive nature of MXenes, their biological impact is inherently dependent on surface chemistry, lateral size, oxidation state, and formulation context.

Short‐term in vitro studies generally report favorable hemocompatibility and low cytotoxicity, particularly when surface functionalization reduces nonspecific membrane interactions [79]. However, nanomaterials can exert dual immunological effects, promoting either regenerative macrophage polarization or pro‐inflammatory activation depending on physicochemical parameters.

In orthopedic and implantable systems, biosafety is intrinsically linked to infection control. Hybrid platforms integrating Ti_3_C_2_ MXene with Fe‐based metal–organic framework (Fe‐MOF) nanozymes within 3D‐printed polylactic acid scaffolds demonstrate how MXene‐mediated photothermal effects can synergize with catalytic ROS generation to achieve potent antibacterial activity [94]. Under NIR irradiation, localized surface plasmon resonance (LSPR) enhances electron transfer to Fe‐MOFs, accelerating Fenton‐like reactions and amplifying ROS production.

While such antibacterial activity improves implant success, it simultaneously necessitates careful evaluation of ROS‐mediated host tissue damage. Therefore, defining therapeutic windows for photothermal activation is essential to balance antibacterial efficacy and cytocompatibility.

**FIGURE 7:**
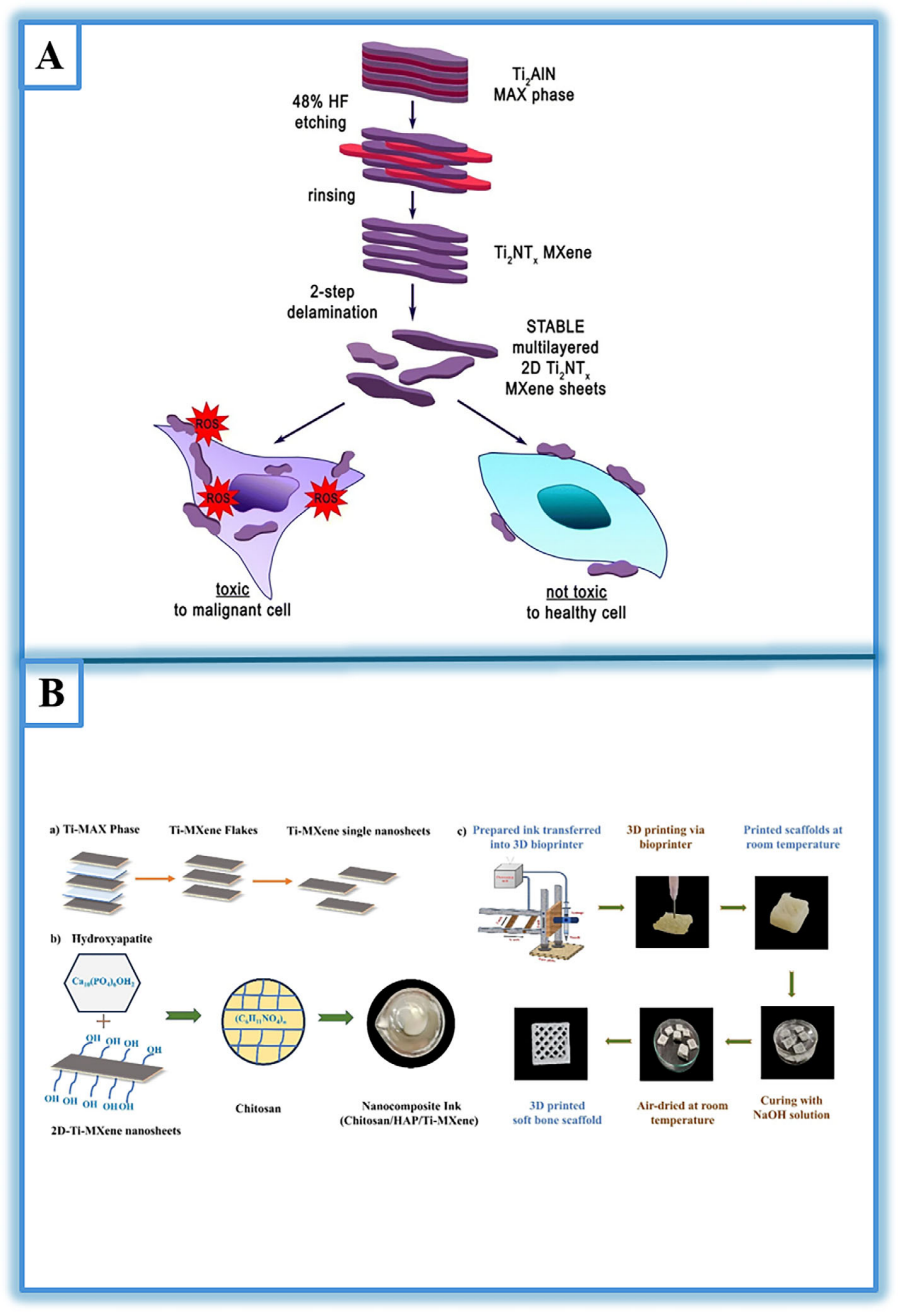
(A) Schematic abstract shows a descriptive workflow outlining the fabrication, synthesis pathway, structural stabilization process, and subsequent interactions with human cells of multilayer 2D Ti_2_NT_x_MXene nanosheets. Reproduced with permission from [155]. Copyright 2019 Springer Nature. (B) Schematic workflow illustrating (a) the synthesis of Ti‐MXene nanosheets, (b) the formulation of chitosan/hydroxyapatite/Ti‐MXene nanocomposite bioink, and (c) the fabrication of 3D‐printed scaffolds for soft bone tissue engineering applications. Reproduced with permission from [159]. Copyright 2025 Nature.

Accumulating evidence indicates that MXene toxicity is highly dose‐ and surface‐dependent [153]. Zebrafish embryo models classify Ti_3_C_2_ as “practically nontoxic” at tested concentrations, with negligible neurodevelopmental disruption [154]. However, differential cytotoxicity toward malignant versus nonmalignant cell lines has been reported, suggesting that oxidation state and surface terminations influence membrane interactions and oxidative stress induction (Figure 7A) [155]. Surface engineering plays a decisive role in mitigating toxicity. Soybean phospholipid‐functionalized Ti_3_C_2_ (Ti_3_C_2_ ‐SP) nanosheets demonstrate minimal cytotoxicity in vitro and no significant acute or chronic toxicity following intravenous administration in murine models at doses up to 20 mg kg^−^^1^ [22]. Similar favorable profiles are observed for SP‐functionalized Ta_4_C_3_ and MnOx/ Ti_3_C_2_ composites, with stable hematological and systemic parameters in vivo [156, 157]. These findings underscore that MXene biosafety is tunable rather than fixed. However, long‐term accumulation, chronic immune activation, and potential genotoxicity remain insufficiently characterized. Standardized nanotoxicology frameworks, aligned with emerging nanomedicine regulatory guidelines, will be required to systematically assess immune profiling, complement activation, and systemic responses [158].

Beyond minimizing toxicity, MXene‐based systems can actively promote constructive host integration. Asaro et al. covalently integrated MXene–collagen biohybrid platforms demonstrate enhanced fibroblast proliferation, improved cardiomyocyte maturation, and antibacterial performance against *Staphylococcus aureus* [108]. Increased connexin‐43 (Cx43) expression under electrical stimulation indicates improved intercellular electrical coupling in cardiac models.

Beyond cardiac and soft‐tissue applications, the favorable biocompatibility and antibacterial properties of MXene‐based systems have also been demonstrated in bone tissue engineering. Veebarathin et al. developed 3D bioprinted chitosan/hydroxyapatite (CS/HAp) nanocomposite scaffolds containing Ti‐MXene to reduce biofilm‐induced infections and improve mechanical performance in bone implants. Chitosan and hydroxyapatite were combined in a 1:1 ratio, and then hydrogels suitable for bioprinting were prepared by adding different concentrations of Ti‐MXene. The results showed that the scaffold containing 0.3 mg/mL Ti‐MXene exhibited better structural integrity, lower biofilm formation, and higher mechanical strength (23.3 MPa). Furthermore, low swelling and degradation rates and favorable surface properties were obtained. Cell viability tests revealed that the scaffold did not exhibit toxic effects and supported cell adhesion. These findings demonstrate that Ti‐MXene‐reinforced nanocomposite scaffolds are biocompatible, antibacterial, and mechanically strong candidates for bone tissue engineering applications (Figure 7B) [159].

These findings illustrate that MXene‐enabled systems operate at the intersection of bioactivity and therapeutic modulation. However, sustained immune equilibrium under chronic implantation conditions remains to be validated through long‐term studies examining fibrosis, foreign body response, and degradation‐mediated immune activation.

### 5.4 Regulatory Classification and Approval Pathways

MXene‐based bioinks occupy a complex and evolving regulatory landscape, where classification is determined by composition, intended clinical indication, and mechanism of action. Depending on whether constructs incorporate living cells, bioactive molecules, drug‐release components, or purely structural scaffolds, MXene‐enabled systems may be categorized as medical devices, biologics, combination products, or advanced therapy medicinal products (ATMPs) under FDA and EMA frameworks [160].

This classification ambiguity introduces substantial translational complexity. Device‐based pathways typically emphasize mechanical performance, biocompatibility, and degradation kinetics, whereas biologic or combination product pathways require extensive pharmacokinetic, biodistribution, immunogenicity, and long‐term safety evaluation. For example, MXene‐based scaffolds designed solely for structural regeneration may follow device‐oriented regulatory routes, while photothermal systems incorporating drug delivery or nitric oxide release mechanisms may be regulated as combination products requiring dual evaluation streams.

Regulatory agencies mandate comprehensive physicochemical characterization for nanomaterial‐containing systems. For MXene‐based bioinks, this includes nanosheet size distribution, surface termination quantification, oxidation stability, batch reproducibility, and degradation product profiling [161].

The dynamic surface chemistry and oxidation susceptibility of Ti_3_C_2_Tx and related MXenes present additional regulatory challenges, as small alterations in termination chemistry can modify biological performance. Therefore, lifecycle stability, from manufacturing through storage and implantation, must be demonstrated under defined quality control parameters.

Three‐dimensional bioprinting further amplifies regulatory complexity. Constructs containing cells and biomolecules often fall within combination or ATMP categories, requiring validation of cell sourcing, sterility assurance, scaffold–cell interaction stability, and functional persistence. Large‐animal preclinical models are increasingly expected to bridge the translational gap by evaluating long‐term tissue integration, vascularization, immune response, and mechanical durability under physiologically relevant loading conditions [79].

A critical challenge lies in the fact that existing regulatory frameworks were not originally designed for multifunctional nanomaterial‐enabled biofabrication platforms [6]. MXenes combine structural, electroactive, photothermal, and biochemical functionalities within a single system, complicating mechanism‐of‐action categorization. Adaptive regulatory strategies, including early scientific advice consultations and risk‐based classification approaches, will therefore be essential. Proactive engagement with regulatory agencies during early development stages can help define testing requirements, reduce uncertainty, and accelerate approval pathways [162].

### 5.5 Ethical, Cost, and Commercial Considerations

Beyond regulatory compliance, the successful clinical adoption of MXene‐based bioinks depends on ethical governance, economic feasibility, and demonstrable clinical value. Particularly in biofabricated systems incorporating living cells, ethical considerations surrounding cell sourcing, donor consent, genetic stability, and long‐term monitoring must be carefully addressed. Autologous cell strategies offer improved immunocompatibility but limit scalability and increase manufacturing complexity, whereas allogeneic approaches improve standardization but introduce immunological and ethical considerations.

Long‐term implantation of electroactive nanomaterials also raises ethical obligations regarding post‐market surveillance and patient risk communication. Given the evolving understanding of nanomaterial biodistribution and chronic exposure effects, transparent risk–benefit assessment and longitudinal monitoring frameworks will be essential for maintaining patient trust and regulatory approval.

From a manufacturing standpoint, cost remains a major barrier. Current MXene synthesis methods involve selective etching processes, controlled delamination, and surface functionalization steps that increase production complexity and variability. Achieving medical‐grade purity with consistent surface termination profiles further elevates cost and demands rigorous quality control. Without scalable and cost‐efficient production pipelines, large‐scale commercialization may remain constrained.

Commercial viability will ultimately depend on clear demonstration of clinical superiority over existing biomaterials. MXene‐based bioinks must justify their added complexity by delivering measurable improvements in functional outcomes, durability, or reduced complication rates. In healthcare systems governed by reimbursement frameworks, cost–benefit analyses and health economic evaluations will strongly influence adoption. Technologies that provide incremental benefits at disproportionately higher costs may struggle to achieve market penetration.

Moreover, intellectual property landscapes and manufacturing know‐how will shape industrial partnerships and technology transfer pathways. Collaborative ecosystems involving academic researchers, material manufacturers, clinicians, and regulatory experts will be required to bridge the gap between laboratory innovation and commercial deployment.

Collectively, ethical responsibility, economic scalability, and demonstrable clinical value represent pivotal determinants of translational success. MXene‐enabled bioinks must therefore be evaluated not only through scientific performance metrics but also through holistic frameworks encompassing societal acceptance, cost‐effectiveness, and sustainable manufacturing strategies [152].

## 6 Challenges and Future Perspectives

Despite rapid advances demonstrating the promise of MXene‐based bioinks for 3D bioprinting, several critical challenges remain before these materials can be broadly applied in functional tissue engineering and clinical settings. Addressing these challenges will require multidisciplinary efforts spanning materials science, bioengineering, regulatory science, and translational research.

Beyond formulation‐related challenges, the design of functionally integrated bioinks introduces additional complexity. MXenes’ exceptional electrical conductivity and surface chemistry can improve bioink performance, but their incorporation also introduces complexity in ink rheology. Achieving uniform MXene dispersion without aggregation is essential for consistent printability, as agglomerates can increase viscosity and lead to nozzle clogging or inhomogeneous filament formation. Meanwhile, strategies to improve flow behavior, such as high‐shear mixing or specific solvents, may inadvertently degrade nanosheet structure or impact biocompatibility, underscoring the delicate balance between printability and functional integrity. Careful optimization of dispersion techniques, potentially guided by machine learning models, could help navigate complex formulation spaces and enhance reliability.

To fully harness MXenes’ capabilities, multi‐component ink designs that synergistically balance mechanical, electrical, and biological functions are an active area of future research. For example, composite formulations that combine MXenes with other conductive fillers, elastomeric polymers, or bioactive ceramics may help maintain printability while providing functional cues tailored to specific tissues, yet such designs require careful engineering to avoid compromising cell viability or structural stability.

A pervasive challenge in 3D bioprinting is the fabrication of constructs that not only mimic the structural complexity of native tissues but also support vascularization and nutrient transport. While MXene‐based bioinks can enhance electrical and mechanical properties, they do not inherently solve the limitations related to creating perfusable microvascular networks that are critical for tissue survival in thick or metabolically active constructs. Future strategies may need to integrate MXene bioinks with sacrificial inks, growth factor gradients, or vascularizing cell populations to emulate functional tissue architecture [163].

Although initial in vitro and in vivo studies indicate good short‐term cytocompatibility for MXene‐based constructs, long‐term biocompatibility remains underexplored. Particularly under physiological conditions, MXenes can undergo oxidation and structural changes that may affect conductivity, functional performance, and degradation byproducts. There is evidence that degradation behavior and immune interactions are strongly dependent on surface functionalization and nanosheet chemistry, but systematic evaluations in long‐term animal models are limited. Addressing these gaps is essential for understanding chronic responses and ensuring safe clinical adoption [4].

Translating MXene bioinks from bench to bedside will require scalable manufacturing processes that ensure batch‐to‐batch reproducibility of nanosheet properties and bioink performance. Current synthesis and functionalization routes for MXenes are often small‐scale and sensitive to etching conditions, making standardization challenging. Regulatory pathways for advanced bioinks incorporating nanomaterials also remain complex, as such constructs may be classified as combination products, requiring extensive safety and efficacy data. Collaboration between researchers and regulatory bodies is needed to establish clear guidelines for nanomaterial‐integrated bioinks, including standardized characterization metrics and evaluation protocols. Future research should focus on predictive and adaptive bioink systems, where MXene properties can be dynamically tuned to match tissue‐specific requirements and evolving biological environments. In the long term, MXene‐based bioinks may evolve from passive structural materials into intelligent biointerfaces capable of sensing, responding, and actively guiding tissue regeneration [134, 164].

Importantly, overcoming these challenges will require not only incremental improvements but also integrated design strategies that bridge material science, biology, and clinical needs. MXene‐based bioinks are emerging as a transformative class of materials with the potential to redefine functional biofabrication.

## 7 Conclusion

MXene‐based bioinks have emerged as a highly versatile and promising class of materials at the intersection of nanotechnology and biofabrication. Their unique combination of physicochemical properties, including high electrical conductivity, tunable surface chemistry, photothermal responsiveness, and mechanical reinforcement capability, enables the design of multifunctional bioinks that extend beyond traditional structural roles. As highlighted throughout this review, these features position MXenes as key enablers for engineering bioactive microenvironments that can actively regulate cell behavior and tissue development.

From a materials engineering perspective, significant progress has been made in tailoring MXene‐based bioinks through advanced formulation strategies, cross‐linking mechanisms, and rheological optimization to achieve printability without compromising biological performance. The ability to integrate electrical conductivity and stimuli‐responsive behavior within printable systems has opened new opportunities for fabricating constructs that mimic the dynamic and functional nature of native tissues.

Biologically, MXene‐incorporated constructs have demonstrated promising outcomes across both in vitro and in vivo studies. These systems support cell viability, proliferation, and lineage‐specific differentiation, while also enabling enhanced functional maturation in electrically active tissues such as muscle and cardiac systems. In vivo evidence further indicates that MXene‐based scaffolds can promote tissue regeneration, modulate immune responses, and facilitate functional integration within host environments. However, these biological effects are highly dependent on material parameters such as surface chemistry, concentration, and degradation behavior, underscoring the importance of precise material design.

Despite these advances, several critical challenges remain to be addressed before clinical translation can be realized. These include achieving reproducible large‐scale synthesis, ensuring long‐term stability under physiological conditions, understanding degradation pathways and biodistribution, and establishing comprehensive biosafety profiles. In addition, regulatory classification, manufacturing standardization, and ethical considerations will play a defining role in determining the translational trajectory of MXene‐based bioinks.

Looking forward, the future of MXene‐enabled biofabrication lies in the development of intelligently engineered systems that integrate structural, electrical, and biological functionalities within a single platform. Emerging approaches combining MXenes with bioactive molecules, responsive polymers, and advanced printing strategies are expected to further expand their applicability across complex tissue systems. Moreover, the integration of predictive modeling and data‐driven design frameworks may accelerate the optimization of MXene‐based bioinks for specific clinical applications.

In conclusion, MXene‐based bioinks represent a transformative direction in 3D bioprinting, offering the potential to bridge the gap between engineered constructs and functional living tissues. Continued interdisciplinary efforts will be essential to fully harness their capabilities and translate these advanced materials from experimental platforms into clinically relevant solutions for regenerative medicine.

## Conflicts of Interest

The authors declare no conflicts of interest.

## Data Availability

The data that support the findings of this study are available from the corresponding author upon reasonable request.
